# Genomic analysis of the Phalaenopsis pathogen *Dickeya* sp. PA1, representing the emerging species *Dickeya fangzhongdai*

**DOI:** 10.1186/s12864-018-5154-3

**Published:** 2018-10-29

**Authors:** Jingxin Zhang, John Hu, Huifang Shen, Yucheng Zhang, Dayuan Sun, Xiaoming Pu, Qiyun Yang, Qiurong Fan, Birun Lin

**Affiliations:** 1 Key Laboratory of New Techniques for Plant Protection in Guangdong, Institute of Plant Protection, Guangdong Academy of Agricultural Sciences, Guangzhou, 510640 China; 2 Department of Plant and Environmental Protection Sciences, College of Tropical Agriculture and Human Resources, University of Hawaii, Honolulu, HI 96822 USA; 3 Department of Plant Pathology, University of Florida, Gainesville, FL 32611 USA

**Keywords:** Comparative genomics, *Dickeya*, Novel species, Secretion systems, CRISPR, PKs/NRPs

## Abstract

**Background:**

*Dickeya* sp. strain PA1 is the causal agent of bacterial soft rot in *Phalaenopsis*, an important indoor orchid in China. PA1 and a few other strains were grouped into a novel species, *Dickeya fangzhongdai*, and only the orchid-associated strains have been shown to cause soft rot symptoms.

**Methods:**

We constructed the complete PA1 genome sequence and used comparative genomics to explore the differences in genomic features between *D*. *fangzhongdai* and other *Dickeya* species.

**Results:**

PA1 has a 4,979,223-bp circular genome with 4269 predicted protein-coding genes. *D*. *fangzhongdai* was phylogenetically similar to *Dickeya solani* and *Dickeya dadantii*. The type I to type VI secretion systems (T1SS–T6SS), except for the *stt*-type T2SS, were identified in *D*. *fangzhongdai*. The three phylogenetically similar species varied significantly in terms of their T5SSs and T6SSs, as did the different *D*. *fangzhongdai* strains. Genomic island (GI) prediction and synteny analysis (compared to *D*. *fangzhongdai* strains) of PA1 also indicated the presence of T5SSs and T6SSs in strain-specific regions. Two typical CRISPR arrays were identified in *D*. *fangzhongdai* and in most other *Dickeya* species, except for *D*. *solani*. CRISPR-1 was present in all of these *Dickeya* species, while the presence of CRISPR-2 varied due to species differentiation. A large polyketide/nonribosomal peptide (PK/NRP) cluster, similar to the zeamine biosynthetic gene cluster in *Dickeya zeae* rice strains, was discovered in *D*. *fangzhongdai* and *D*. *solani*. The *D*. *fangzhongdai* and *D*. *solani* strains might recently have acquired this gene cluster by horizontal gene transfer (HGT).

**Conclusions:**

Orchid-associated strains are the typical members of *D*. *fangzhongdai*. Genomic analysis of PA1 suggested that this strain presents the genomic characteristics of this novel species. Considering the absence of the *stt*-type T2SS, the presence of CRISPR loci and the zeamine biosynthetic gene cluster, *D*. *fangzhongdai* is likely a transitional form between *D. dadantii* and *D*. *solani*. This is supported by the later acquisition of the zeamine cluster and the loss of CRISPR arrays by *D*. *solani*. Comparisons of phylogenetic positions and virulence determinants could be helpful for the effective quarantine and control of this emerging species.

**Electronic supplementary material:**

The online version of this article (10.1186/s12864-018-5154-3) contains supplementary material, which is available to authorized users.

## Background

Pathogens in the genus *Dickeya*, family *Pectobacteriaceae* [1], cause bacterial soft rot disease, with an increased risk of infection observed in diverse host plants worldwide [2]. The classification and taxonomy of this genus are complex [3] and have evolved in recent years. Previously, genetic markers and biochemical tests divided these pathogens into six species: *D. chrysanthemi*, *D*. *dianthicola*, *D*. *dieffenbachiae*, *D*. *paradisiaca*, *D*. *dadantii* and *D*. *zeae* [3, 4]. *D*. *solani*, which is closely related to *D*. *dadantii*, has emerged in recent years and mostly infects potato [5]. Strains found in water rather than in plants define the species *D. aquatica* [6]. Most recently, strains that cause bleeding cankers on pear trees in China have been proposed as a novel species, namely, *D*. *fangzhongdai*, named in honor of Professor Zhongda Fang [7]. Among the previously identified species, *D*. *dieffenbachiae* and *D*. *dadantii* are closely related and *D*. *dieffenbachiae* has been suggested to represent *D*. *dadantii* subsp. *dieffenbachiae* [8].

In Guangdong Province, China, bacterial soft rot caused by *Dickeya* spp. is a serious disease, infecting numerous important crops, including banana [9, 10], rice [11, 12], and *Philodendron* ‘Con-go’ [13]. The most popular homegrown flower in Guangdong, the *Phalaenopsis* orchid (*Phalaenopsis* Blume, 1825), is also threatened by these pathogens. We isolated the *Dickeya* strain PA1 from these orchids. In flower nurseries, *Phalaenopsis* orchids exhibit the typical symptoms of water-soaked, pale to dark brown pinpoint spots on leaves [14]. Since its discovery, the prevalence of this orchid disease has increased and is becoming one of the most devastating diseases in the local flower industry. In other regions of the world, bacterial soft rot is an important disease that affects Orchidaceae plants, and the major pathogens are *Pectobacterium* spp. and *Dickeya* spp., both in the family *Pectobacteriaceae*. Although some *Dickeya* pathogens have been reported on orchids, the species remain unknown [15]. Strains grouped into the undefined *Dickeya* lineages (UDLs) are important components of the causal agents of bacterial soft rot in ornamental plants [16]. *Dickeya* sp. B16 and S1 from these UDLs have also been found to infect orchids and were identified as *D*. *fangzhongdai* [17]. In China, *Dickeya* species, *P. carotovora*, *Burkholderia cepacia* and *Pseudomonas* spp. were previously reported to cause bacterial soft rot among Orchidaceae. Orchid pathogens from the genus *Dickeya* were identified as *D. chrysanthemi* or *D*. *dadantii*; however, these species were probably misidentified due to the evolving classification within the genus. Strain PA1 was previously considered to be *D. dadantii* subsp. *dieffenbachiae* [14], but the exact classification of this strain remains in question.

DNA sequencing is used routinely in pathogen diagnostics and whole-genome sequencing offers the advantage of increased precision of classification. In addition, comparisons of closely related genomes can clarify niche adaptation, partly because of the relatively low level of genetic variation and simplification of both genomic reconstruction and polymorphism [18]. Numerous draft or complete sequences of the genomes of *Dickeya* spp. are available from the National Center for Biotechnology Information (NCBI). These sequences may facilitate functional and comparative genomic studies to determine how the genomes of closely related species have evolved [19]. For this study, we obtained the complete genome of *Dickeya* sp. PA1, an important pathogen in the orchid industry of Guangdong, China. PA1 and several other strains were placed in a newly described species, *D*. *fangzhongdai*, as these strains were distinguishable from representative strains of the other well-characterized *Dickeya* species. The backbone of *Dickeya* is largely conserved, but some genomic variation has contributed to virulence among the species [20]. Thus, we compared the completed genome of strain PA1 to the available genomes of species from the *Dickeya* genus. This comparative genomic study provides a basis for determining the relatedness and evolution of genes and proteins involved in virulence and bacterial differentiation. It also provides insight into the genomic adaptation of closely related strains to different host plants or environments.

## Results and discussion

### Genome assembly and annotation

**Fig. 1:**
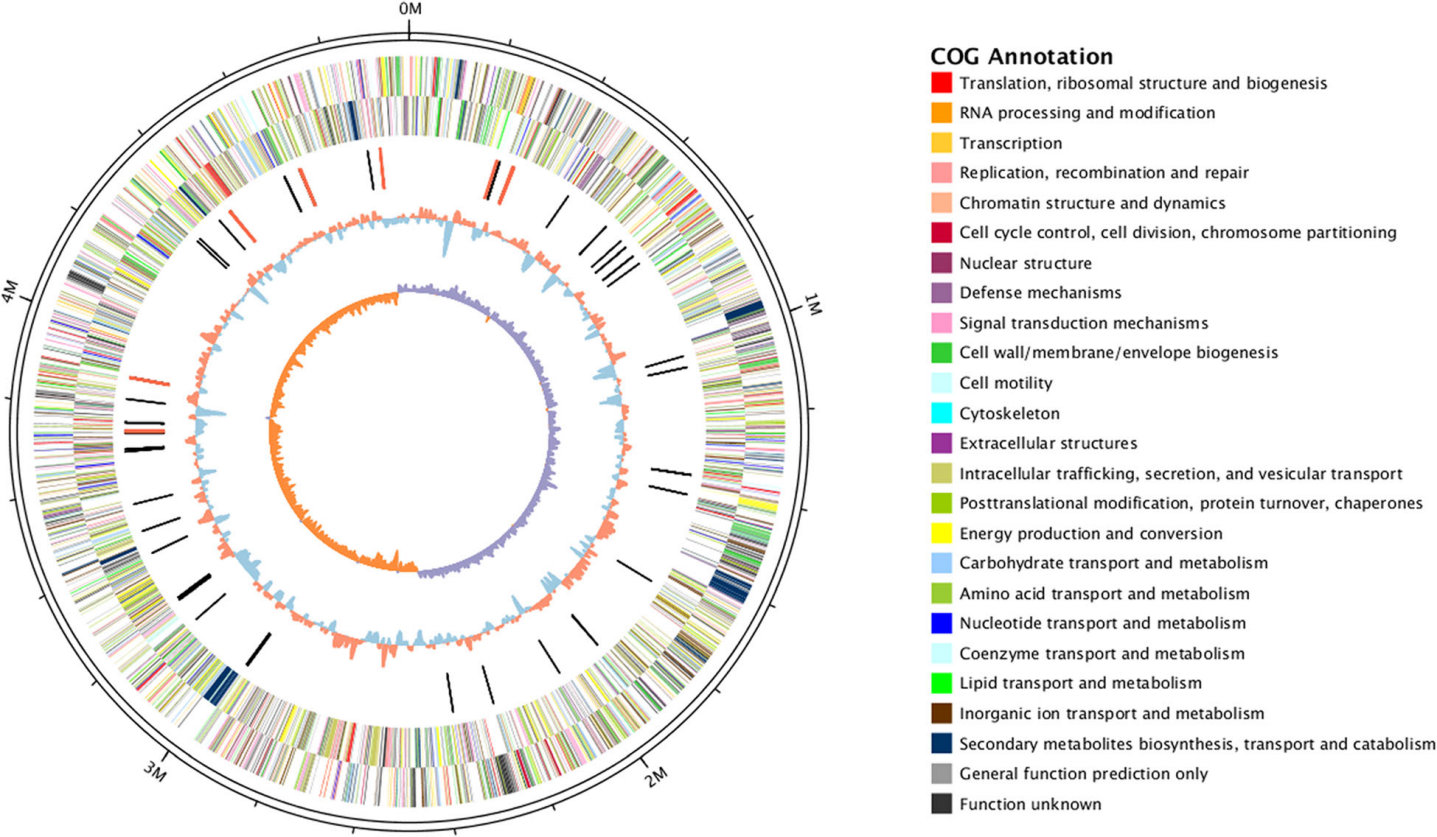
Circular visualization of the complete genome of *D*. *fangzhongdai* PA1. Circles from outside to inside indicate predicted genes in the positive strand, predicted genes in the negative strand, ncRNA (black indicates tRNA, red indicates rRNA), G + C content and GC skew value (GC skew = (G-C)/(G + C); purple indicates > 0, orange indicates < 0)

We predicted 4269 open reading frames (ORFs) within the 4,979,223-bp complete genome sequence of PA1 with 56.88% G + C content. The circular chromosome had an initial *dnaA* start codon; information on the predicted gene distribution, clusters of orthologous groups of proteins (COG) annotation, and G + C content are indicated in Fig. 1. Within this chromosome, we found 75 tRNA genes and 7 rRNA regions. In the predicted rRNA regions, two and four common organization types (16S–23S-5S) were present on the positive and negative strands, respectively. An unusual organization type (16S–23S-5S-5S) was also found on the negative strand. The complete genome of PA1 was deposited in GenBank under accession no. CP020872.

### Phylogenetic characterization of PA1 using housekeeping genes and whole-genome sequences

**Fig. 2:**
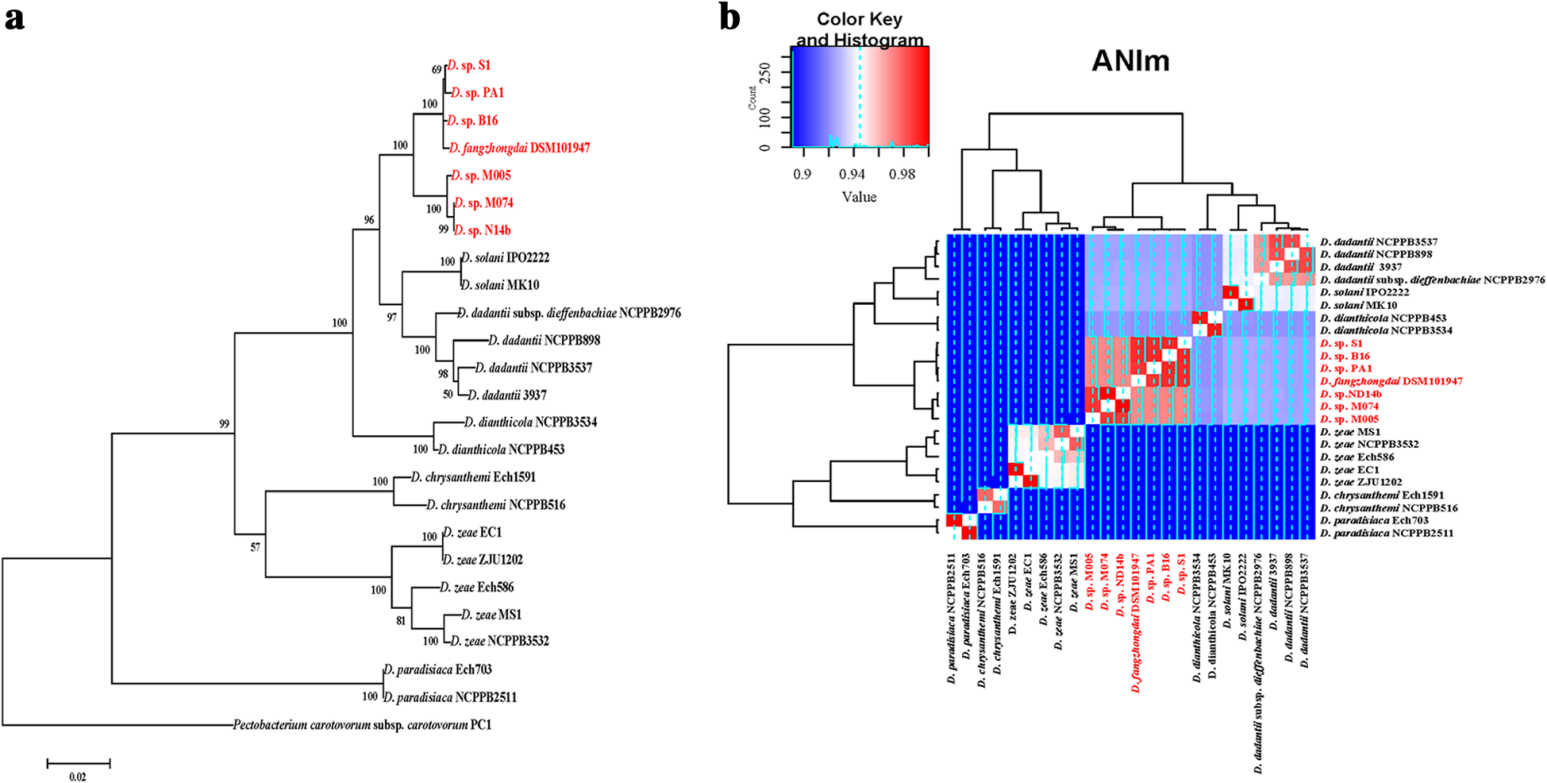
Phylogenetic characterization of *D*. *fangzhongdai* PA1. **a** Phylogenetic analysis of *Dickeya* strains from different species based on concatenated sequences of the genes *dnaX*, *recA*, *dnaN*, *fusA*, *gapA*, *purA*, *rplB*, *rpoS* and *gyrA*. Confidence values on the branches were obtained with Mega 5.1, bootstrapped at 1000 replicates. Twenty-four *Dickeya* strains, including strain PA1, were used for phylogenetic analyses. **b** ANI analysis based on the complete or draft genomes of *Dickeya* strains

Phylogenetic analysis separated the well-identified *Dickeya* species into different clades using both housekeeping genes and genome sequences (Fig. 2a, Additional file 1). PA1 was grouped into a clade with two strains isolated from orchids, *Dickeya* spp. S1 and B16 from Slovenia. In the same clade was a strain isolated from a pear tree, *D*. *fangzhongdai* DSM101947 from China, and three strains isolated from waterfalls, *Dickeya* spp. ND14b, M005 and M074 from Malaysia [21]. In this novel clade, hereafter designated *D*. *fangzhongdai*, the plant strains were close in phylogenetic distance and were separated from the Malaysian waterfall strains. Notably, only PA1, B16 and S1 were reported as causal agents of soft rot and all were isolated from diseased orchids. Average nucleotide identity (ANI) analysis and in silico DNA-DNA hybridization (isDDH) (formula 2) showed that the seven *D*. *fangzhongdai* strains were different from other well-characterized species (Fig. 2b, Additional file 2). ANI values among pairs of these seven strains were greater than 0.97, and ANI values among each of these seven strains and strains of the other well-characterized species were 0.84–0.93. These data facilitated the classification of the strains PA1, S1, B16, ND14b, M005 and M074 as new members of the species *D*. *fangzhongdai*, as the suggested cutoff for species delineation is 0.96 for the ANI value [22] and 0.7 for the isDDH value [21].

**Fig. 3:**
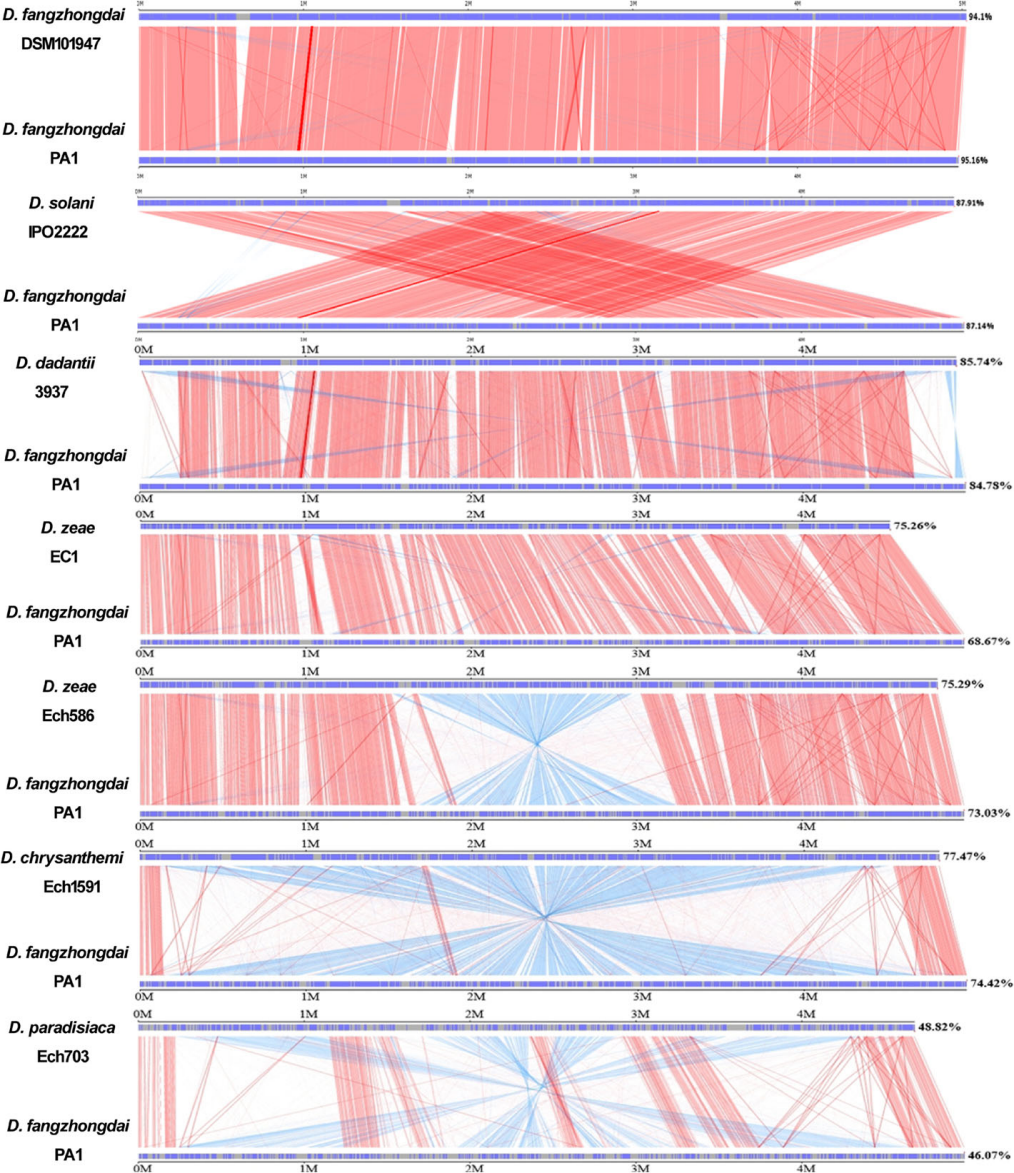
Linearity analysis between *D*. *fangzhongdai* PA1 and other *Dickeya* strains based on whole-genome sequences. The strains used have complete genome sequences, and linearity analysis was performed based on a nucleic acid sequence BLAST. Red indicates homologous regions present in the same orientation; blue indicates homologous regions present in inverted orientation

Synteny analysis indicated that *D*. *fangzhongdai* PA1 shared a high collinearity with *D*. *solani* IPO2222 (87.91%) and *D. dadantii* 3937 (85.74%) (Fig. 3). ANI values between the *D*. *fangzhongdai* strains and either *D. dadantii* or *D*. *solani* strains were 0.92–0.93 (Fig. 2b). These findings both indicated that *D*. *fangzhongdai* was closely related to *D. dadantii* and *D*. *solani*. Notably, the isDDH values between the strains of *D. fangzhongdai* and either *D*. *solani* or *D*. *dadantii* were greater than 0.70 when formula 1 or 3 was used (Additional file 2). Different molecular and biochemical analyses all indicated that *D*. *solani* was closely related to *D*. *dadantii* [5]. Thus, *D. fangzhongdai*, *D. dadanti*i and *D*. *solani* are likely genetically related, making bacterial differentiation complicated during the evolution of these three species.

### Genomic dissimilarities between PA1 and other closely related *D. fangzhongdai* strains

Twenty genomic islands (GIs) were predicted from the complete genome of *D. fangzhongdai* PA1, and the genes associated with these GIs were also identified (Additional files 3, 4). These findings included the following genes: genes encoding rearrangement hotspot (Rhs) proteins in the type VI secretion system (T6SS) gene cluster; the *phnI*–*phnL* gene cluster associated with phosphonate metabolism; two response receiver proteins and two histidine kinases in a two-component system; and a large exoprotein of the hemolysin BL-binding protein in the T5SS (Additional file 4). Synteny analysis indicated that PA1 was highly collinear with *D. fangzhongdai* DSM101947, B16, and S1, with similarities of 94.10% (Fig. 3), 96.57% and 95.59%, respectively. The comparison between PA1 and these three strains revealed small differences among the wide range of virulence factors. For example, T4SS, the filamentous hemagglutinin of T5SS, and the Rhs proteins of T6SS were identified in regions of PA1 that were unmatched in the other three strains. Therefore, it was possible to explore the genomic characteristics of the novel species *D. fangzhongdai* using strain PA1 as the representative strain.

### Conserved features of T1SS–T4SS in *Dickeya* species

Similar to other *Dickeya* strains [23], the T1SS of *D. fangzhongdai* PA1 consisted of three proteins: PrtD (*B6N31_11100*), PrtE (*B6N31_11095*) and PrtF (*B6N31_11090*). Four metalloproteases, PrtG, PrtB, PrtC and PrtA (*B6N31_11110*, *B6N31_11085*–*B6N31_11075*), were found adjacent to PrtD and PrtF. Another group of extracellular enzymes, namely, the plant cell-wall-degrading enzymes, were also conserved in the PA1 genome. Most of these enzymes were secreted through the T2SS. An *out*-type T2SS encoded by *outS* and a 13-gene operon (*outB*–*outO*) was present in PA1 (Additional file 5). The genes *outS* and *outB*–*outM* were conserved in the genomes of all identified *Dickeya* species. Another T2SS operon consisting of *sttD*–*sttM* and *sttS*, is present in the chromosomes of *D*. *dadantii* subsp. *dadantii*, *D*. *dadantii* subsp. *dieffenbachiae*, *D. chrysanthemi* and *D*. *dianthicola*. This second *stt*-type T2SS was not found in the available genomes of *D. fangzhongdai* or in the genomes of *D*. *zeae*, *D*. *solani* or *D*. *paradisiaca*.

For the T3SS encoded by a cluster that included the *hrp* (hypersensitive response and pathogenicity) and *hrc* (hypersensitive response conserved) genes [24], *D. fangzhongdai* PA1 harbored a large 29-gene operon, spanning a genomic region of approximately 27.4 kb (Additional file 6). The *hrp*/*hrc* cluster was also present in the genomes of most *Dickeya* species, except *D*. *paradisiaca*. The core *hrp* and *hrc* gene clusters were highly conserved, with only a few differences present in the near-upstream region of the *plcA* gene. The *D*. *fangzhongdai* gene cluster encoding the effector DspE was similar to the one observed in the closely related species *D*. *dadantii* and *D*. *solani*, except for a gene encoding a PKD protein present only in *D. fangzhongdai* (Additional file 7).

In the PA1 genome, the *virB*-T4SS gene cluster consisting of *virB1*, *virB2*, *virB4*–*virB11*and *kikA*, spanned a genomic region of approximately 10.2 kb (Additional file 8). Among the different *Dickeya* species, this was absent in *D*. *paradisiaca*. VirB4 is found in most bacterial species [25, 26] and VirB4 homologs were conserved within the *Dickeya* strains harboring the T4SS apparatus. A few T4SSs in gram-negative bacteria and most T4SSs in gram-positive bacteria lack VirB11 homologs [25, 26]. VirB11 was present in all *Dickeya* strains carrying the T4SS gene cluster. However, unlike the other *D. fangzhongdai* strains, DSM101947 did not contain the T4SS gene cluster.

### Variations in the flagellar-type T3SS were greater than those in the *hrp*-type T3SS

**Fig. 4:**
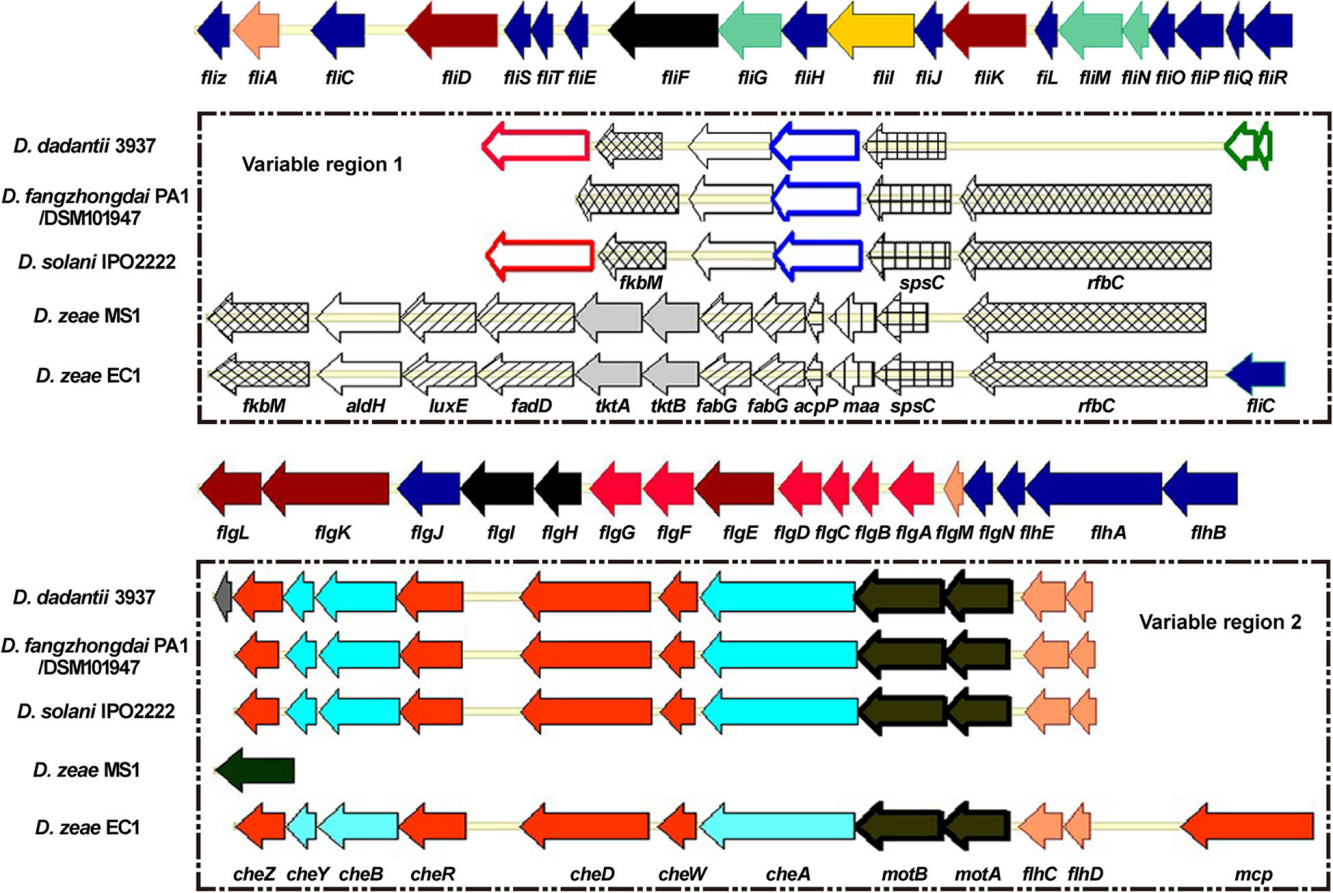
Genomic organization of the flagellar-type T3SS in *Dickeya* strains. The flagellar-type T3SS in *D*. *fangzhongdai* PA1 is at locus *B6N31_13995*–*B6N31_14255*. Variable region 1 is located between the loci of *fliA* and *fliC*; variable region 2 extends from the locus of *flhB* to the end of the *fli* and *che* clusters. Variable region 2 in *D. zeae* MS1 is at locus *J417_RS0103870*–*J417_RS0104170*.  flagellar protein and flagellin;  transcription factor;  flagellar hook-associated protein;  flagellar ring protein;  flagellar motor switch protein;  ATP synthase;  flagellar basal-body protein;  methyltransferase;  oxidoreductase;  fatty acid synthase;  transketolase;  acyl carrier protein;  maltose O-acetyltransferase;  aminotransferase;  carbamoyl-phosphate synthase;  transposase;  chemotaxis protein;  chemotaxis family TCS;  flagellar motor protein;  integrase

The flagellar apparatus is considered a subtype of the T3SS and was characterized in the chromosomes of *Dickeya* strains. The *D. fangzhongdai* PA1 chromosome had a complete set of flagellar genes, spanning 53.6 kb. These genes encoded flagellin FliC, 39 flagellar biosynthesis proteins, 2 flagellar motor proteins and 7 chemotaxis-associated proteins (Fig. 4). The *fli* and *che* clusters were also present in other *Dickeya* species, including *D*. *paradisiaca* Ech703 (*Dd703_1507*–*Dd703_1559*) and NCPPB 2511 (*DPA2511_RS07770*–*DPA2511_RS08030*). This latter result differed from the results of Zhou et al. [27]. The typical gene arrangement, *fliC*–*fliD*–*fliR*, found in most *Dickeya* species was not observed in *D*. *paradisiaca* strains, which had a *fliC*–*fliR*–*fliD* orientation. The *D. fangzhongdai* strains, including PA1, were similar to other *Dickeya* species, including *D. solani* IPO2222, *D. dadantii* 3937, *D*. *zeae* EC1 and MS1 at this locus, except for two variable regions (Fig. 4, Additional file 9).

In variable region 1, located between the *fliA* and *fliC* genes, all *D. fangzhongdai* strains except B16 had a *rfbC* methyltransferase gene similar to that of *D. solani* IPO2222, while *D. dadantii* 3937 had two transposase genes instead (Fig. 4). At the same locus, this methyltransferase also was found in three other Guangdong strains: *D*. *zeae* MS1, which infects banana; *D*. *zeae* EC1 and ZJU1202, which infect rice, as well as another *D*. *zeae* rice strain DZ2Q (GenBank accession NZ_APMV00000000.1) from Italy. However, within variable region 1 these four strains also contained an additional gene cluster (*luxE*-*fadD*-*tktA*-*tktB*-*fabG*-*fabG*) encoded fatty acid biosynthesis components (Fig. 4, Additional file 9). This cluster was absent in other *D. zeae* genomes. The presence of this gene cluster in banana strain MS1 indicated a genetic exchange among *D*. *zeae* strains infecting different nearby hosts.

The variable region 2, which extended from *flhB* to the end of the *fli* and *che* clusters, contained duplicates of the *fhlDC*, *motA*/*B* and seven chemotaxis-associated genes. While conserved in different *Dickeya* species, this region was absent in *D. zeae* MS1, where it was replaced by a cluster containing integrase genes at each end and phage genes in between (Fig. 4, Additional file 9).

The presence of transposase genes in *D. dadantii* 3937 and of a fatty acid biosynthesis cluster in the *D*. *zeae* MS1 and the rice strains, plus the replacement of the *fli* and *che* genes by integrase genes in MS1, suggested a potentially active locus for genetic exchange around the *fli* and *che* clusters in *Dickeya* strains. In *D. fangzhongdai* strains, variable region 2 was conserved, but there was significant variation in variable region 1. B16 lacked the large methyltransferase gene, similar to *D. dadantii* 3937, and the *D*. *fangzhongdai* Malaysian strains ND14b, M005 and M074 lacked *fkbM*.

### Variations in the T5SS among *D*. *fangzhongdai* strains and strains from closely related species

**Fig. 5:**
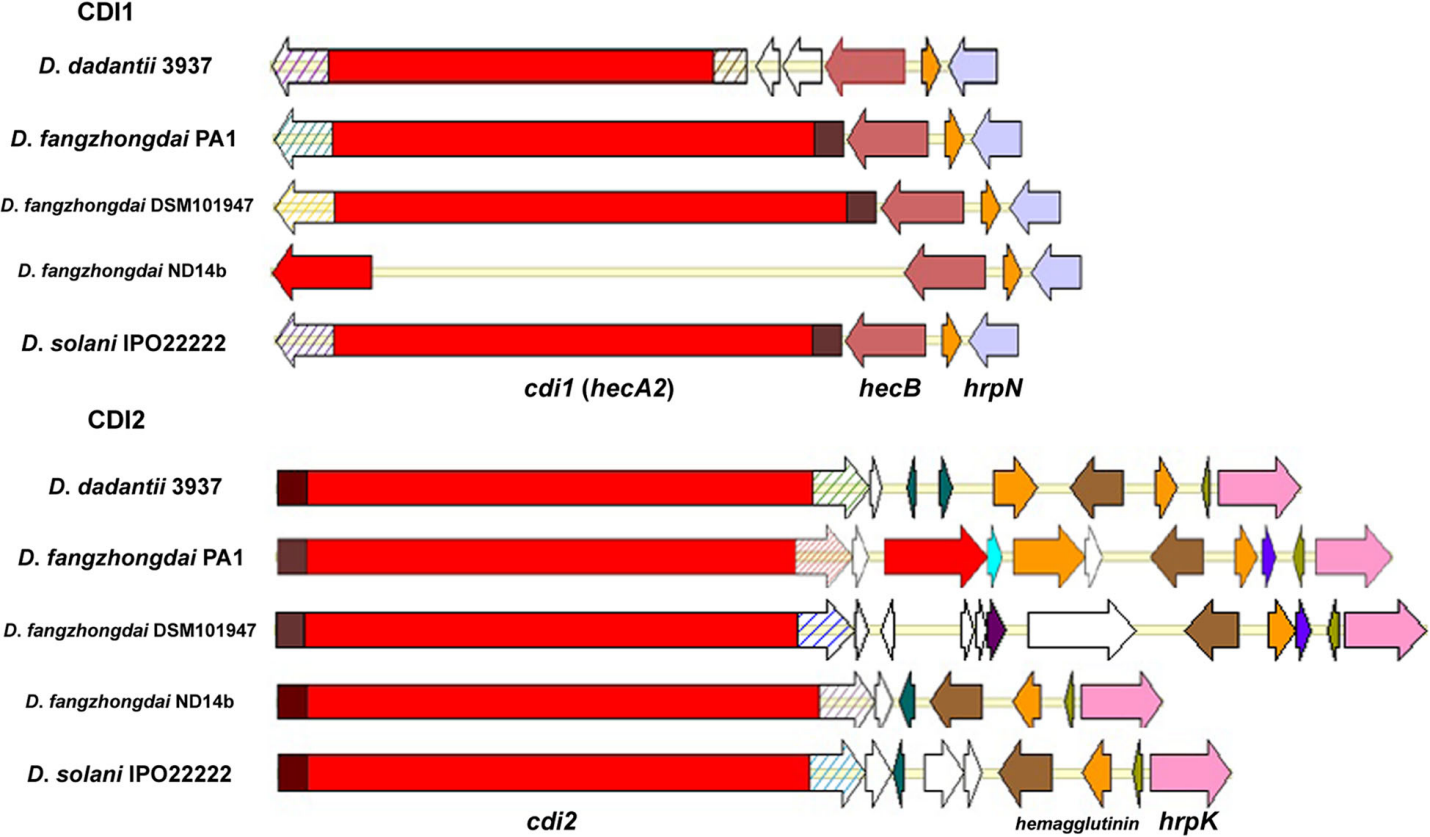
Genomic organization of the T5SS in *Dickeya* strains. The *cdi1* and *cdi2* genes in *D. fangzhongdai* PA1 are at loci *B6N31_12005* and *B6N31_11515*, respectively. Homologous gene domains are shown in the same color. The N-terminal and C-terminal toxin domains are shown individually, and diagonal lines indicate divergent domains

Compared with the other secretion systems, T5SS has the simplest genomic features [28] and is the least explored. The T5SS consists of either one or two proteins, the latter constituting a two-partner secretion (Tps) system. The Tps system consists of TpsB (HecB) and the large effector protein TpsA (HecA2). The genes *hecA2* and *hecB* were found in proximity to T3SS in the chromosome of *D. fangzhongdai* PA1 (Fig. 5). This Tps system may be found among various necrogenic plant pathogens, even those lacking a T3SS, since these pathogens encode at least one HecA2 homolog [29]. Among the *Dickeya* strains analyzed, HecB was present in all genomes, indicating that the Tps system was also universal among these *Dickeya* strains. However, we found that the gene sequences encoding HecA2 in some *Dickeya* strains were incomplete. This finding may have been due to genomic variations that resulted in truncated protein translation, as observed in *D*. *fangzhongdai* ND14b (Fig. 5). Alternatively, the complicated genome structures with high G + C content in this region may have interrupted the sequencing process, as observed with other incomplete genomes.

Tps systems in *Dickeya* spp. participate in contact-dependent growth inhibition (CDI) [30]. HecA2 is designated CdiA (Cdi1 in this study) and is involved in CDI, in which target bacterial cells are bound by the delivery of a C-terminal toxin domain (Cdi-CT). Two CDI systems were identified in *D. fangzhongdai*, *D*. *solani* and *D*. *dadantii*, and Cdi1 and Cdi2 were respectively the major CDI proteins in these two systems (Fig. 5). We also predicted a DNA/RNA nonspecific endonuclease domain in PA1-Cdi2 similar to the one observed in 3937-Cdi2. Comparison of the N termini (110 aa) of Cdi1 and Cdi2 of *D*. *fangzhongdai* PA1 with other homologous proteins of the strains in the most closely related species, *D*. *solani* IPO2222 and *D*. *dadantii* 3937, and other strains in *D*. *fangzhongdai*, revealed that these domains in *D*. *fangzhongdai* PA1 were conserved among *Dickeya* strains, excluding *D*. *dadantii* 3937-Cdi1. A hemagglutination activity domain, hemagglutinin repeat, and a pretoxin domain with a VENN motif were predicted from the Cdi analog in *Dickeya*. However, hemagglutination activity domains were absent in some strains with nonconserved N termini, such as 3937-Cdi1. In contrast, Cdi-CTs diverged greatly among Cdi analogs. Additionally, the genomic organization of the CDI2 cluster of strain PA1 was different from that of the CDI2 clusters of those close related strains. PA1 contained an additional *cdi* gene (*B6N31_11495*) that was homologous with *cdi1* and *cdi2*. *D*. *fangzhongdai* DSM101947 and ND14b lacked the additional *cdi* homologous gene, as did IPO2222 and 3937. Moreover, *D*. *fangzhongdai* ND14b harbored an incomplete *cdi1* gene in the CDI1 locus and had only the CDI2 system (Fig. 5). Thus, there was greater variation in T5SS than in T1SS –T4SS among the closely related species *D*. *dadantii*, *D*. *solani* and *D*. *fangzhongdai* and even among *D*. *fangzhongdai* strains, in addition to the prevalence of T5SS. The variation included the diversity of both N-terminus domains and CdiA-CTs and the difference of genomic organization of CDI proteins.

### Variations in the T6SS among *D*. *fangzhongdai* strains and strains from closely related species

**Fig. 6:**
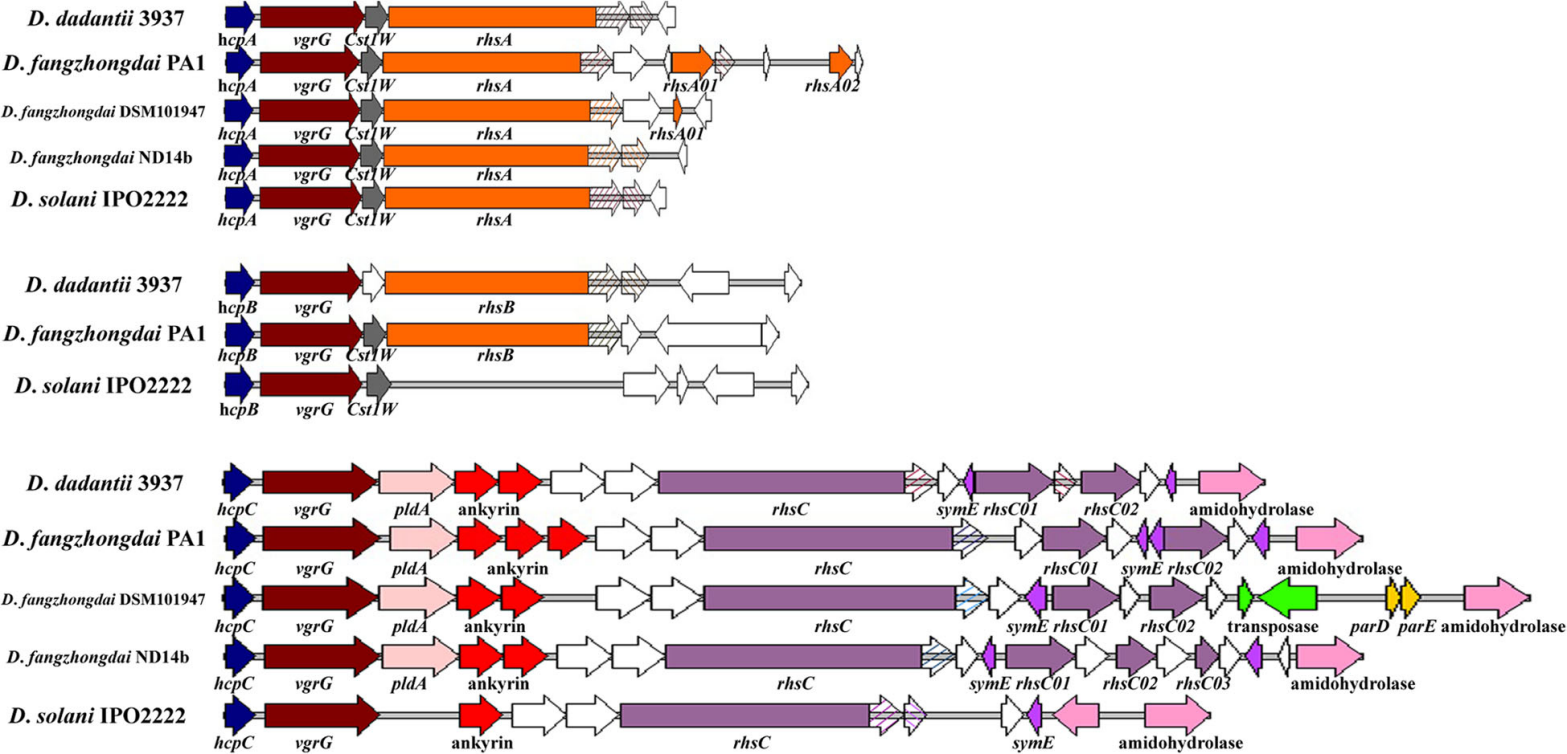
Genomic organization of the T6SS in *Dickeya* strains. The three *hcp* genes in *D*. *fangzhongdai* PA1 are at loci *B6N31_00250*, *B6N31_08590*, *B6N31_07000*. Homologous gene domains are presented in the same color and diagonal lines indicate divergent domains

The hemolysin-coregulated protein (Hcp) and valine-glycine repeat protein G (VgrG) are the external components of the T6SS [31]. Our results showed that Hcp and VgrG were conserved in most *Dickeya* strains, excluding strains of *D*. *paradisiaca*. The number of these protein pairs varied among the strains of closely related species: *D*. *fangzhongdai* PA1, *D. dadantii* 3937 and *D. solani* IPO2222 contained three pairs; *D*. *fangzhongdai* DSM101947 and *D*. *fangzhongdai* ND14b each contained two pairs. The three *rhs* genes and gene clusters found in the chromosome of PA1 were all linked to the corresponding *hcp* and *vgrG* genes, as were those in *D. dadantii* 3937 (Fig. 6). However, in other *Dickeya* strains the *hcp* and *vgrG* genes were not always linked to the *rhs* genes in T6SS [32]. *D. solani* IPO2222 contained a *rhsA* locus downstream of *hcpA* rather than *hcpB*. In *D*. *fangzhongdai*, DSM101947 and ND14b had only *hcpA* followed by *rhsA* in these two genomic regions (Fig. 6). Phylogenetic analysis of the RhsA/B protein sequences suggested that *D*. *fangzhongdai* PA1 was more closely related to *D. dadantii* 3937, while *D*. *fangzhongdai* DSM101947 and ND14b were more closely related to *D. solani* IPO2222 (Additional file 10). Given this observation and the copy numbers of the Rhs toxin systems, the *D*. *fangzhongdai* strains exhibited distinct differentiation. PA1 was more similar to *D*. *dadantii* 3937, while DSM101947 and ND14b were more similar to *D*. *solani* IPO2222.

In addition to variations in the number of Rhs toxin systems in the most closely related species, the strains also differed in the toxin/antitoxin moiety of the C-terminal toxin domains (Rhs-CT). Consistent with previous studies [33, 34], the Rhs proteins RhsA, RhsB and RhsC in *D*. *fangzhongdai* PA1 and other *Dickeya* strains encoded large conserved N-terminal domains that included YD peptide repeats and had extensive polymorphisms in the Rhs-CT (Fig. 6). These repeats were analogous to the hemagglutinin repeats of Cdi proteins [23, 34].

The gene cluster of *rhsC* was the only locus with a whole set of T6SS-secreted proteins. Phylogenetic analysis of the Rhs protein sequences suggested a close relationship between the RhsA and RhsB proteins, but the RhsC proteins were grouped in an independent cluster (Additional file 10). At the *rhsC* locus, the core genes encoding the T6SS-secreted proteins ImpB, ImpC, lysozyme, ImpG, ImpH, filamentous hemagglutinin, VasD, ImpJ, ImpK, ClpB, Sfa, VasI, ImpA, ImpA, IcmF and VasL (*B6N31_07090*–*B6N31_07160*) were conserved in different *Dickeya* species, but these genes were absent in *D*. *paradisiaca*. Additional toxin-immunity modules are always arranged in tandem arrays downstream of the main *rhs* genes [23], and the closely related *Dickeya* species, *D*. *fangzhongdai*, *D. dadantii* and *D. solani* also differed in the number and sequences of additional orphan *rhs* toxin-immunity pairs at the *rhsC* locus. *D*. *fangzhongdai* PA1 and *D. dadantii* 3937 had two additional *rhs* genes, and *D. solani* IPO2222 had only the main *rhsC* gene. In *D*. *fangzhongdai*, DSM101947 had two additional *rhs* genes at the *rhsC* locus, as did PA1, but ND14b contained three additional *rhs* genes. Moreover, *D*. *fangzhongdai* PA1 had two additional *rhs* toxin-immunity pairs at the *rhsA* locus and DSM101947 had one. The other *D*. *fangzhongdai* strains, as well as *D. dadantii* 3937 and *D. solani* IPO2222, lacked these additional genes (Fig. 6). These orphan toxin-immunity pairs appeared to be horizontally transferred between bacteria, so the variance among these pairs also may have contributed to the structural diversity of toxins in the different strains [23]. Thus, the phylogenetically similar strains varied significantly in their genomic organization of toxin effectors and additional orphans in T6SS and the polymorphisms of their toxin domains, similar to T5SS. Additionally, the copy numbers of the toxin systems of T6SS could have varied among these strains.

### Distinctive clustered regularly interspaced short palindromic repeat (CRISPR) types

**Fig. 7:**
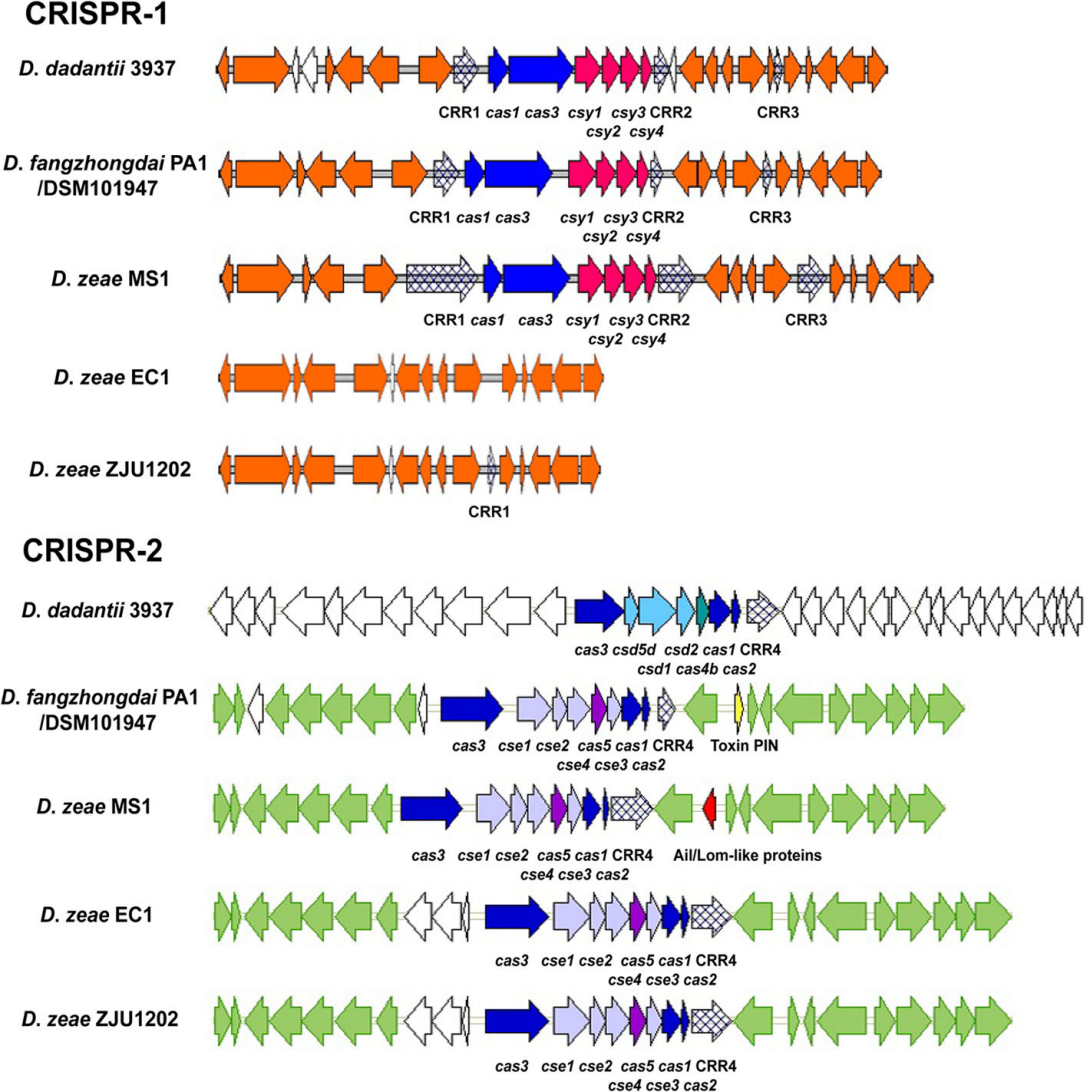
Genomic organization of CRISPRs in *Dickeya* strains. Homologous gene domains are shown in the same color. The toxin PIN gene in *D*. *fangzhongdai* PA1 is at locus *B6N31_0200*. The Ail/Lom family gene in *D*. *zeae* MS1 is at locus *J417_RS0102360*

Virulence factors associated with secretion systems play a role in bacterial differentiation and host-specific pathogenesis. However, genomic hypervariation also can be caused by selection pressure or by the genomic activity of specific phages [35]. CRISPRs are widely distributed and found in 40% of bacteria and almost all archaea [36] and can protect an organism against bacteriophages and foreign plasmids [37]. Genomic analysis revealed two distinct types of direct repeat (DR) analogs in the CRISPR sequences of *Dickeya* genomes. There were no CRISPR sequences in the typical strains of *D. solani*. The first type of DR analog (GTNNACTGCCGNNNAGGCAGCTTAGAAA) array was present in all CRISPR-harboring species. *D*. *fangzhongdai* strains and the other CRISPR-harboring strains, excluding *D*. *zeae* rice strains, contained a CRISPR subtype I-F array named CRISPR-1. It consisted of CRISPR-associated (Cas) core proteins (Cas1, Cas3) and Csy proteins (Csy1–Csy4) (Fig. 7). The *D*. *zeae* rice strains ZJU1202 and DZ2Q contained one simple DR sequence array that lacked Cas and Csy proteins, and EC1 lacked the whole array (Fig. 7). Subtype I-F CRISPR-1 was universally present in *Dickeya* strains and might have been present in ancestral strains of *Dickeya* spp. The CRISPR-1 array would have been acquired by horizontal gene transfer (HGT) before the differentiation of *Dickeya* strains. This hypothesis was strongly supported by the presence of phage-related genes or gene clusters upstream and downstream of CRISPR-1.

The second type of DR sequence was found in combination with the core proteins Cas1, Cas2 and Cas3, constituting CRISPR-2 (Fig. 7). However, the DR sequences varied depending on the species. In the first group, homologous DR sequences were observed in *D*. *fangzhongdai*, *D. dadantii* subsp. *dieffenbachiae*, *D. zeae*, and *D*. *paradisiaca*, constituting the CRISPR subtype I-E array, CRISPR-2, with the Cas core proteins and Cse proteins (Cse1–Cse4) (Fig. 7). In the second group, *D*. *dadantii* subsp. *dadantii* and *D*. *dianthicola* contained other types of homologous DR sequences. These sequences were arranged with *csd* and *cas* genes (CRISPR subtype I-C) at a different locus than in the first group (Fig. 7). *D. chrysanthemi* belonged to the third group, and most strains of this species, excluding NCPPB402, also had another subtype I-E CRISPR-2 array with a different DR sequence at another genomic position. This diverse CRISPR-2 may have been acquired during species differentiation [38]. Therefore, CRISPR-2 could be used to differentiate among *Dickeya* species, especially to distinguish strains of *D. dadantii* subsp. *dieffenbachiae* from the most closely related subspecies *D. dadantii* subsp. *dadantii*. Surprisingly, we found the toxin PIN inside the subtype I-E CRISPR-2 array in the *D*. *fangzhongdai* Chinese and European strains and in *D. dadantii* subsp. *dieffenbachiae* strains. We also found an Ail/Lom family protein at the same locus in *D*. *zeae* MS1, Ech586 and NCPPB3532 (Fig. 7). Members of this family with Ail/Lom-like proteins are known virulence factors of *Yersinia enterocolitica* [39]. Clarification of the function of the toxin PIN and Ail/Lom family proteins in CRISPR-2 requires further investigation.

Differentiation at the CRISPR-2 locus was relatively high among the *D*. *fangzhongdai* strains. Unlike the *D*. *fangzhongdai* Chinese and European strains, the *D*. *fangzhongdai* Malaysian strains did not harbor the toxin PIN homolog. Notably, the DR sequences at CRISPR-2 in *D. dadantii* subsp. *dieffenbachiae*, *D. zeae*, and *D*. *paradisiaca* were the same, but the sequences in *D*. *fangzhongdai* B16, ND14b and M074 varied at one or two base pairs. Nevertheless, DR sequences are generally highly conserved throughout the locus [40]. Thus, *D*. *fangzhongdai* was more similar to *D*. *dadantii* at the CRISPR loci. These DR sequence variations indicate that under selection pressure *D*. *fangzhongdai* might have undergone repeat degeneration [41, 42]. This may have resulted in the loss of CRISPR sequences, however, such as in *D*. *solani*.

### A nonribosomal peptide (NRP) and polyketide (PK) cluster similar to the zeamine biosynthetic gene cluster

**Fig. 8:**
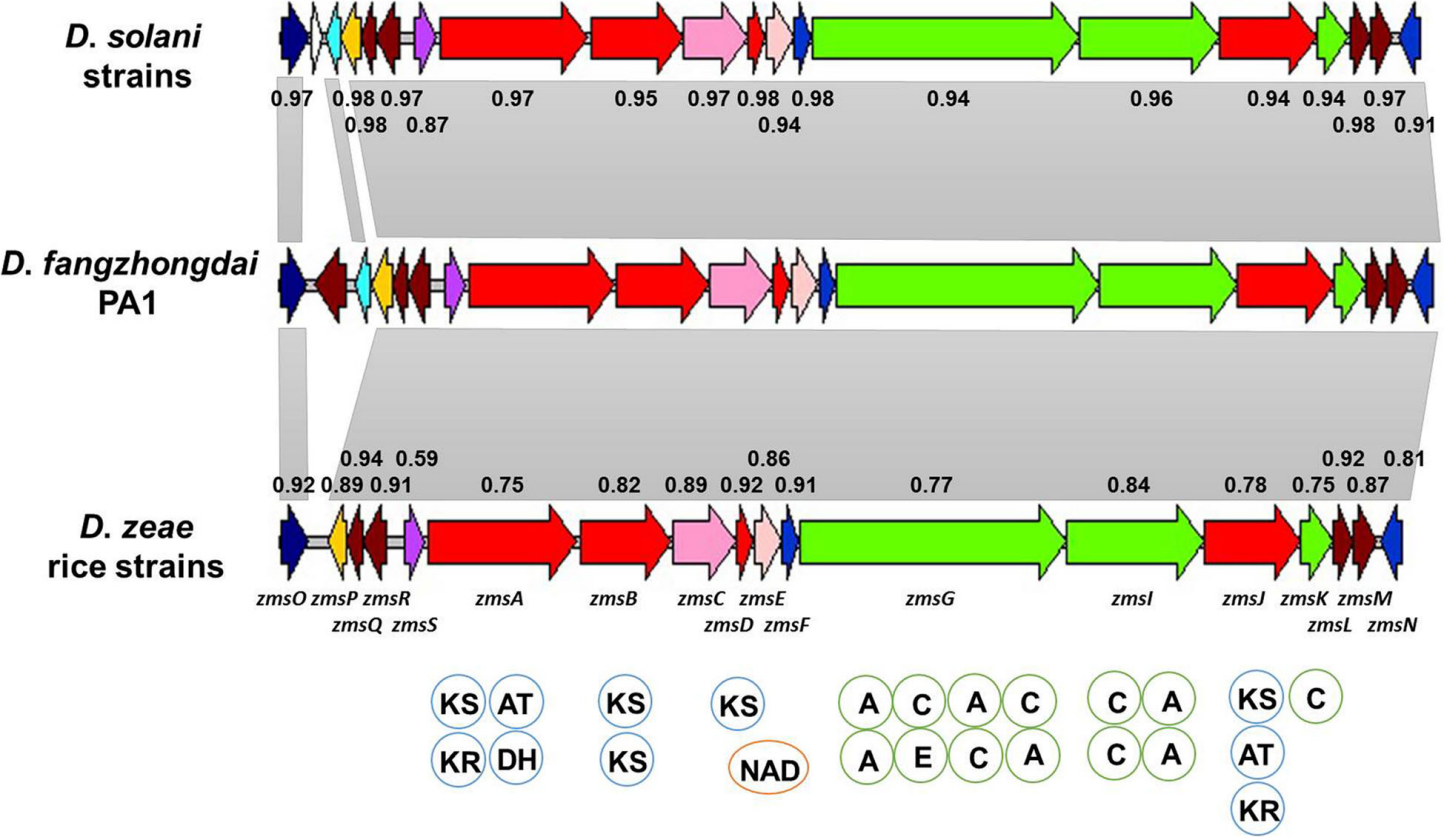
Genomic organization of the homologous zeamine biosynthetic gene clusters in *Dickeya* strains. Genes *zmsO* and *zmsP*–*zmN* in *D*. *fangzhongdai* PA1 are at loci *B6N31_07205* and *B6N31_07230*–*B6N31_07310*, respectively. Genes aligned with a shadow were homologous, and the numbers indicate the percentages of identity of each protein compared with homologous proteins in *D*. *fangzhongdai* PA1.  peroxidase;   ABC transporter;  secretion protein HlyD;  membrane protein;  polyketide synthase;  polyunsaturated fatty acid synthase;  thioester reductase;  hydrolase;  nonribosomal peptide synthase;  phosphopantetheinyl transferase;  hypothetical protein. Circles = the domain predicted in PK or NRP genes. KS = keto reductase domain; AT = acyl transferase domain; KR = keto reductase domain; DH = dehydratase domain; NAD = NAD-binding domain; A = AMP-binding domain; C = condensation domain; E = epimerization domain. The PK domains include KS, AT, KR and DH, and the NRP domains include A, C and E

Many microorganisms produce bioactive secondary metabolites, such as antibiotics, anticancer agents and other substances [43]. PKs and NRPs are two representative classes of enzymes that synthesize important secondary metabolites [44]. We found a large gene cluster similar to the zeamine biosynthetic gene cluster from *Serratia plymuthica* in *D*. *fangzhongdai* PA1. The core genes of this cluster included *zmsO* and *zmsP*–*zmN* (Fig. 8). The proteins encoded by these core genes were highly conserved among *D*. *fangzhongdai* strains (percentages of identity of 97–99%). Homologous gene clusters were found in *D*. *fangzhongdai*, *D*. *solani* and the *D*. *zeae* rice strains, but not in other species (Fig. 8). The same linear arrangement of functional domains was predicted from the arrangement of the encoded proteins (Fig. 8).

The Zms proteins of *D*. *fangzhongdai* PA1 had a higher identity with *D*. *solani* Zms proteins (91–98%, except 87% for the the ZmsS protein encoding fatty acid synthase) than with the proteins in the *D*. *zeae* rice strains (75–94%, except 59% for the ZmsS) (Fig. 8). PA1 and the *D*. *zeae* rice strains were somewhat related regarding genes encoding NRPs and PKs. In *D*. *zeae* rice strain EC1, the zeamine biosynthesis gene cluster plays a key role in bacterial virulence [27]. Therefore, the role of this gene cluster in the pathogenesis of *D*. *fangzhongdai* and *D*. *solani* strains needs to be explored in the future. Alternatively, the G + C content of the homologous *zms* gene clusters in *D*. *fangzhongdai* and *D*. *solani* strains was approximately 61%. This was significantly higher than the G + C contents of the whole genomes of the *D*. *fangzhongdai* strains (56.60–56.88%) and the *D*. *solani* strains (56.10–56.40%). These findings indicated that the gene clusters in the *D*. *fangzhongdai* and *D*. *solani* strains might have been acquired recently via HGT during bacterial differentiation. The presence of the zeamine biosynthetic gene cluster homolog in *D*. *fangzhongdai* indicated that this newly described species was more similar to *D*. *solani* at this locus. Further, this gene cluster might be a unique phytopathogenicity determinant [45] differentiating these species from the *D*. *dadantii*.

Three additional PK/NRP clusters located at previously predicted GIs were expected in the *D*. *fangzhongdai* PA1 genome (Additional file 4). An NRP-TransatPK type PK/NRP gene cluster was present in GI13. In this cluster, 50% of the genes were similar to the known biosynthetic gene cluster of *Pseudomonas costantinii* secreting tolaasin, which is a virulence factor [46]. This gene cluster was not conserved in the *Dickeya* genus and was found only in some strains of *D*. *fangzhongdai* (PA1, M005), *D*. *solani* (IPO2222, MK10) and *D*. *dadantii* (NCPPB 898). A second NRP-type cluster similar to a gene cluster known to encode enterobactin, was predicted in GI14. This gene cluster was more conserved in different *Dickeya* species. Finally, a third NRP-type gene cluster was identified in GI20. Homology analysis predicted related biosynthetic gene clusters only in *D*. *fangzhongdai* PA1, DSM101947, S1 and B16, and not *D*. *fangzhongdai* Malaysian strains or in the other *Dickeya* species.

### *D*. *fangzhongdai* Chinese and European strains were closely related

We divided strains of the novel species *D*. *fangzhongdai* into three subpopulations according to their geographical distribution. The Chinese subpopulation included strains PA1 and DSM101947; the European subpopulation included B16, S1 and MK7; and the Malaysian subpopulation included ND14b, M005 and M074. To determine genetic differentiation among the different subpopulations, concatenated sequences of nine housekeeping genes, concatenated sequences of *zmsO* and *zmsP*–*zmN* or genomic sequences of the zeamine gene cluster, were tested by three statistical methods, Ks*, Z, and Snn, using DNA Sequence Polymorphism v5 (DnaSP v5) software. These tests indicated a higher divergence between the Malaysian subpopulation and the other two subpopulations than occurred between the other two subpopulations (Additional file 11). *F*_ST_ is the interpopulational component of genetic variation, or the standardized variance in allele frequencies across populations. We used it to measure the level of gene flow between subpopulations as calculated by DnaSP v5. An absolute value of *F*_ST_ < 0.33 suggests frequent gene flow [47]. *F*_ST_ values among the Chinese and European subpopulations were less than 0.33, indicating frequent gene flow. However, gene flow between the Malaysian subpopulation and the other two subpopulations was infrequent (> 0.33) (Additional file 11). The Chinese and European subpopulations both contained pathogens affecting the local orchid industry and these two regions frequently trade in orchids. This might explain the transport of pathogens between these regions. To reduce the spread of this disease in the orchid industry and potentially the entire ornamental plant industry [15], inspection for and quarantine of this pathogen is the best strategy. Therefore, the establishment and phylogenetic determination of the novel species *D*. *fangzhongdai* and classification of the important orchid pathogen PA1 in China, is important for controlling this disease.

## Conclusions

The phylogenetic determination of *D*. *fangzhongdai* PA1 is needed for effective quarantine and pathogen control legislation worldwide. It is especially important in the ornamental plant trade, since the pathogenic strains of this novel species frequently infect orchids. Comparative genomic analyses of *D*. *fangzhongdai*, represented by strain PA1, revealed that the T5SS, T6SS, CRISPR array and PK/NRP loci were present in regions with genomic variation distinct from those of other closely related *Dickeya* species, such as *D. dadantii* and *D*. *solani*. The absence of an *stt*-type T2SS and the presence of the CRISPR arrays and zeamine biosynthetic gene cluster in *D*. *fangzhongdai* might imply that this novel species represents a transitional form between *D. dadantii* and *D*. *solani*. This hypothesis is supported further by the later acquisition of the zeamine cluster and loss of the CRISPR arrays in *D*. *solani*. Comparative genomic analyses provided important insight into phytopathogenicity determinants and their genetic relationships with closely related species and will assist the development of future control strategies for emerging pathogens.

## Methods

### Bacterial culture and genomic DNA extraction

Bacteria were grown to a concentration of 10^8^ CFU/mL in LB medium (10 g/L Bacto tryptone, 5 g/L yeast extract, and 10 g/L NaCl; pH 7.0) in an incubator shaker at 100 rpm and 30 °C. Total DNA was extracted from a 2-mL bacterial suspension using the TIANamp Bacterial DNA Kit (Tiangen Biotech, Beijing, China) according to the manufacturer’s directions.

### Whole-genome sequencing of the *Dickeya* sp. strain PA1

For whole-genome sequencing of strain PA1, we used both paired-end sequencing with the GS FLX+ Titanium platform (Roche, Basel, Switzerland) at Macrogen (Seoul, South Korea) and long-read sequencing with the PacBio RS II sequencing platform (Pacific Biosciences, Menlo Park, United States) at Genedenovo (Guangzhou, China). Construction and sequencing of a GS FLX+ shotgun library was performed by following the standard protocols recommended by Roche. Prior to the elimination of primer concatemers, sequences with weak signal and poly-A/T tails and vector sequences were removed from the raw sequences using SeqClean. Low-quality reads were then trimmed and repeat sequences screened using RepeatMasker [48]. These procedures yielded 208,204 reads with an average length of 808 bp, totaling 168,305,557 bases. High-quality reads were assembled in a Newbler assembler (version 2.6), yielding a draft genome of 64 contigs, each over 500 bp. The draft genome of strain PA1 was 4,916,490 bp in length (approximately 34.23 × coverage of the genome) with 56.85% G + C content.

For long-read sequencing with PacBio RSII, SMRTbell DNA libraries with fragment sizes > 10 kb were prepared from fragmented genomic DNA after g-tube treatment and end repair. SMRT sequencing was performed using P4-C2 chemistry according to standard protocols. Long reads were obtained from three SMRT sequencing runs and those longer than 500 bp with a quality value exceeding 0.75 were merged into a single dataset. Random errors in long-seed reads (seed-length threshold of 6 kb) were corrected with a hierarchical genome assembly process [49] by aligning shorter reads against the long-seed reads. These procedures yielded 82,402 reads with an average length of 8139 bp, amounting to 670,677,839 bases. The corrected reads were used for de novo assembly with the Celera assembler and an overlap-layout-consensus strategy [50]. The Quiver consensus algorithm [49] was then used to validate the quality of the assembly and determine the final genome sequence. The ends of the assembled sequence were trimmed, producing a circular genome of 4,979,223 bp (approximately 134.70 × coverage of the genome) with 56.88% G + C content. The closed genome was corrected for sequencing errors using reads generated by the GS FLX+ platform.

### Annotation of the *Dickeya* sp. strain PA1 genome

We conducted genome annotation by first predicting ORFs and RNAs. ORF prediction was performed with GeneMarkS [51], a well-studied gene-finding program used for prokaryotic genome annotation. Repetitive elements, noncoding RNAs, and tRNAs were searched for with RepeatMasker [48], rRNAmmer [52], and tRNAscan [53], respectively. We then used a combination of complementary approaches for functional annotation by applying BLAST against the NCBI nonredundant protein (Nr) database, UniProt/Swiss-Prot, Kyoto Encyclopedia of Genes and Genomes (KEGG), Gene Ontology (GO), COG, and protein families (Pfam) databases with an E-value cutoff of 1 × 10^− 5^. The predicted genes in the positive and negative strands, COG annotation, tRNA, rRNA, G + C content and GC skew value (GC skew = (G-C)/(G + C)) are presented in a circular layout constructed using Circos [54].

### Phylogenetic characterization of *Dickeya* sp. strain PA1

We selected 24 strains (Table 1) from the *Dickeya* genus for which complete nucleotide sequences of the genes *dnaX*, *recA*, *dnaN*, *fusA*, *gapA*, *purA*, *rplB*, *rpoS* and *gyrA* were available [5]. The *Dickeya* strains used for this analysis included type *Dickeya* strains from a previous study [55]. *Pectobacterium carotovorum* subsp. *carotovorum* PC1 was used as an outgroup control. A preliminary automatic alignment of the sequences was generated using ClustalW [56] with a gap penalty of 15, followed by clipping to the lengths of the consensus sequences. The concatenated sequences of these nine genes were used for phylogenetic analysis by MEGA 5.1 [57] and a maximum likelihood algorithm bootstrapped with 1000 replications. OrthoMCL v.1.4 was used to extract the protein sequences of each bacterial strain (Table 1) and determine the orthologous relationships of the proteins [58]. OrthoMCL was run with a BLAST E-value cutoff of 1 × 10^− 5^, a minimum aligned sequence length coverage of 50% of the query sequence, and an inflation index of 1.5. We retrieved orthologous groups present as single copies from all of the genomes. Phylogenetic analysis using concatenated nucleic acid sequences was performed on the RAxML webserver with maximum likelihood inference bootstrapped with 1000 replicates after gene alignment and filtering of low-quality alignments, as described by Zhang et al. [21]. Pairwise ANI and isDDH values were also obtained from Zhang et al. [21] with the minor modification that formulas 1–3 were used for the isDDH calculation. The isDDH value independent of genome lengths calculated by formula 2 has been recommended for analyses of incomplete genomes [22, 59] and was applied for species definition in this study.

**Table 1:** General features of *Dickeya* strains and genomes used for genomic comparison

Strain	Species	Genome length (Mb)	G + C %	Complete/draft genome	Isolated from	Geographic origin	Year of isolation
PA1 (CP020872)	*D*. *fangzhongdai*	4.98	56.88	Complete	*Phalaenopsis* orchid	Guangdong, China	2011
DSM101947 (CP025003^a^)		5.03	56.79	Complete	*Pyrus pyrifolia*	Zhejiang, China	2009
B16 (NZ_JXBN00000000)		4.89	56.70	Draft	*Phalaenopsis* orchid	Slovenia	2010
S1 (NZ_JXBO00000000)		4.89	56.80	Draft	*Phalaenopsis* orchid	Slovenia	2012
ND14b (CP015137)		5.05	56.90	Complete	Waterfall	Malaysia	2013
M005 (NZ_JSXD00000000)		5.11	56.60	Draft	Waterfall	Malaysia	2013
M074 (NZ_JRWY00000000)		4.95	56.80	Draft	Waterfall	Malaysia	2013
3937 (CP002038)	*D. dadantii* subsp. *dadantii*	4.92	56.30	Complete	*Saintpaulia ionantha*	France	1977
NCPPB3537 (CM001982)		4.81	56.48	Draft	*Solanum tuberosum*	Peru	
NCPPB898 (CM001976) (T^b^)		4.94	56.30	Draft	*Pelargonium capitatum*	Comoros	1960
NCPPB2976 (CM001978) (T)	*D. dadantii* subsp. *dieffenbachiae*	4.8	56.41	Draft	*Dieffenbachia* sp.	USA	1977
IPO2222 (CP015137)	*D. solani*	4.92	56.20	Complete	*Solanum tuberosum*	Netherlands	2007
MK10 (CM001839)		4.94	56.20	Draft	*Solanum tuberosum*	Israel	
Ech1591 (CP001655)	*D. chrysanthemi*	4.81	54.50	Complete	*Zea mays*	USA	1957
NCPPB516 (CM001904)		4.62	54.24	Draft	*Parthenium argentatum*	Denmark	1957
NCPPB3534 (CM001840)	*D. dianthicola*	4.87	55.64	Draft	*Solanum tuberosum*	Netherlands	1987
NCPPB453 (CM001841)(T)		4.68	55.95	Draft	*Dianthus caryophyllus*	UK	1956
Ech703 (CP001654)	*D. paradisiaca*	4.68	55.00	Complete	*Solanum tuberosum*	Australia	
NCPPB2511 (CM001857)(T)		4.63	55.00	Draft	*Musa paradisiaca*	Colombia	1970
EC1 (CP006929)	*D. zeae*	4.53	53.40	Complete	*Oryza.sativa*	Guangdong, China	1997
ZJU1202 (NZ_AJVN00000000)		4.59	53.30	Draft	*Oryza.sativa*	Guangdong, China	2012
MS1 (NZ_APWM00000000)		4.75	53.30	Draft	*Musa* sp.	Guangdong, China	2009
Ech586 (CP001836)		4.82	53.60	Complete	*Philodendron* Schott	USA	
NCPPB 3532 (CM001858)		4.56	53.60	Draft	*Solanum tuberosum*	Australia	

^a^indicates GenBank accessions

^b^indicates the type strains

### Genomic comparison of *Dickeya* bacterial genomes

Annotations of whole-genome sequences of some typical strains of *Dickeya fangzhongdai* and six other well-characterized species were retrieved from the NCBI database and used for genomic comparison (Table 1).

GIs in the complete genome of strain PA1 were predicted using IslandViewer 3. This program integrates the GI prediction methods SIGI-HMM, IslandPath-DIMOB and IslandPick [60]. Synteny analysis was performed using Mummer (https://github.com/mummer4/mummer). CRISPRFinder [61] was used to search for CRISPRs among the completed or draft genome sequences of the *Dickeya* strains by predicting the sequences of DRs and spacers. PKs and NRPs of the *Dickeya* strains were predicted with antiSMASH 3.0 [62]. To compare known virulence factors, we conducted a BLAST search to identify genes common to or specific among all the *Dickeya* genomes available at NCBI with an E-value threshold of 1 × 10^− 5^ and an identity cutoff of 80%.

## Additional files

Additional file 1: Phylogenetic analysis of *Dickeya* strains based on complete or draft genome sequences. Orthologous groups present as a single copy in all of the analyzed *Dickeya* species were retrieved and their concatenated nucleic acid sequences used for phylogenetic analysis. (PDF 222 kb)

Additional file 2: Calculation of isDDH between the genome sequences of *D*. *fangzhongdai* PA1 and other *Dickeya* strains. The GGDC web service (http://ggdc.dsmz.de/ggdc.php) uses three specific distance formulas (formulas 1–3) to calculate single genome-to-genome distance values; formula 2 is recommended for incomplete genomes. (XLSX 13 kb)

Additional file 3: GIs of *D*. *fangzhongdai* PA1 according to IslandViewer. This program integrates two sequence composition GI prediction methods, SIGI-HMM and IslandPath-DIMOB, and a single comparative GI prediction method, IslandPick. GIs predicted by one or more tools are highlighted in red on the outer circle and indicated by numbers. (TIF 4659 kb)

Additional file 4: A functional prediction of the genes encoded by each GI predicted in the PA1 genome. Twenty GIs were predicted from the PA1 genome. Genomic regions indicate the exact genomic positions of each GI. Locus tags indicate genes encoded within the genome sequences of each GI. The predicted COG functions indicate the functional annotation based on the COG database. PK/NRP clusters were predicted within GI13, GI14 and GI20, including *B6N31_13175*–*B6N31_13415*, *B6N31_15010*–*B6N31_15240*, *B6N31_21480*–*B6N31_21670*. (XLSX 13 kb)

Additional file 5: Genomic organization of *out*-type T2SS in *Dickeya* strains. The *out*-type T2SS in *D*. *fangzhongdai* PA1 is at locus *B6N31_15160*–*B6N31_15235*. Blank boxes = T2SS component proteins; *outS* = gene encoding lipoprotein; *outO* = gene encoding prepilin peptidase. (TIF 498 kb)

Additional file 6: Genomic organization of *hrp*-type T3SS in *Dickeya* strains. The *hrp*-type T3SS in *D*. *fangzhongdai* PA1 is at locus *B6N31_12160*–*B6N31_12020*. (TIF 2625 kb)

Additional file 7: Genomic organization of effector gene clusters of the T3SS in *Dickeya* strains. The effector gene cluster of the T3SS in *D*. *fangzhongdai* PA1 is at locus *B6N31_11415*–*B6N31_11450*. DspE/F = Avr family protein; HrpW = type III secreted protein; OrfC = DNA-binding protein; HrpK = pathogenicity locus protein. (TIF 1739 kb)

Additional file 8: Genomic organization of *vir* clusters of the T4SS in *Dickeya* strains. The *vir* cluster of the T4SS in *D*. *fangzhongdai* PA1 is at locus *B6N31_08470*–*B6N31_08520*. (TIF 1366 kb)

Additional file 9: Synteny analysis of *Dickeya* strains based on sequences of the flagellar-type T3SS. Strains analyzed included *D*. *fangzhongdai* PA1, DSM101947 and ND14b; *D*. *dadantii* 3937; *D*. *solani* IPO2222; and *D*. *zeae* EC1 and MS1. Nonconserved regions are indicated by different-colored frames. (TIF 7041 kb)

Additional file 10: Phylogenetic analysis of *D*. *fangzhongdai*, *D*. *dadantii* and *D*. *solani* strains based on the protein sequences of RhsA, RhsB and RhsC in T6SS. (PDF 156 kb)

Additional file 11: Genetic differentiation and gene flow analysis of different *D*. *fangzhongdai* subpopulations. The Chinese subpopulation of *D. fangzhongdai* included PA1 and DSM101947. The European subpopulation included B16, S1 and MK7. The Malaysian subpopulation included ND14b, M005 and M074. Nucleotide diversity (Pi) indicates the average number of nucleotide variations per site. Ks*, Z, and Snn were all calculated for statistical testing of differentiation among different subpopulations. *F*_ST_ was measured to determine the level of gene flow between subpopulations; an absolute value of *F*_ST_ < 0.33 suggests frequent gene flow. (XLSX 11 kb)

## Abbreviations

ANI: Average nucleotide identity
CDI: Contact-dependent growth inhibition
CRISPR: Clustered regularly interspaced short palindromic repeat
DR: Direct repeat
GI: Genomic island
Hcp: Hemolysin-coregulated protein
HGT: Horizontal gene transfer
isDDH: In silico DNA-DNA hybridization
NRP: Nonribosomal peptide
PK: Polyketide
Rhs: Rearrangement hotspot
T1SS–T6SS: Type I–type VI secretion systems
Tps: Two-partner secretion
VgrG: Valine-glycine repeat protein G
