# Supplementary figures and images for: Genomic analysis of the Phalaenopsis pathogen Dickeya sp. PA1, representing the emerging species Dickeya fangzhongdai
Source: BMC Genomics. 2018 Oct 29;19:782. doi: 10.1186/s12864-018-5154-3 (PMC6206727; doi:10.1186/s12864-018-5154-3)

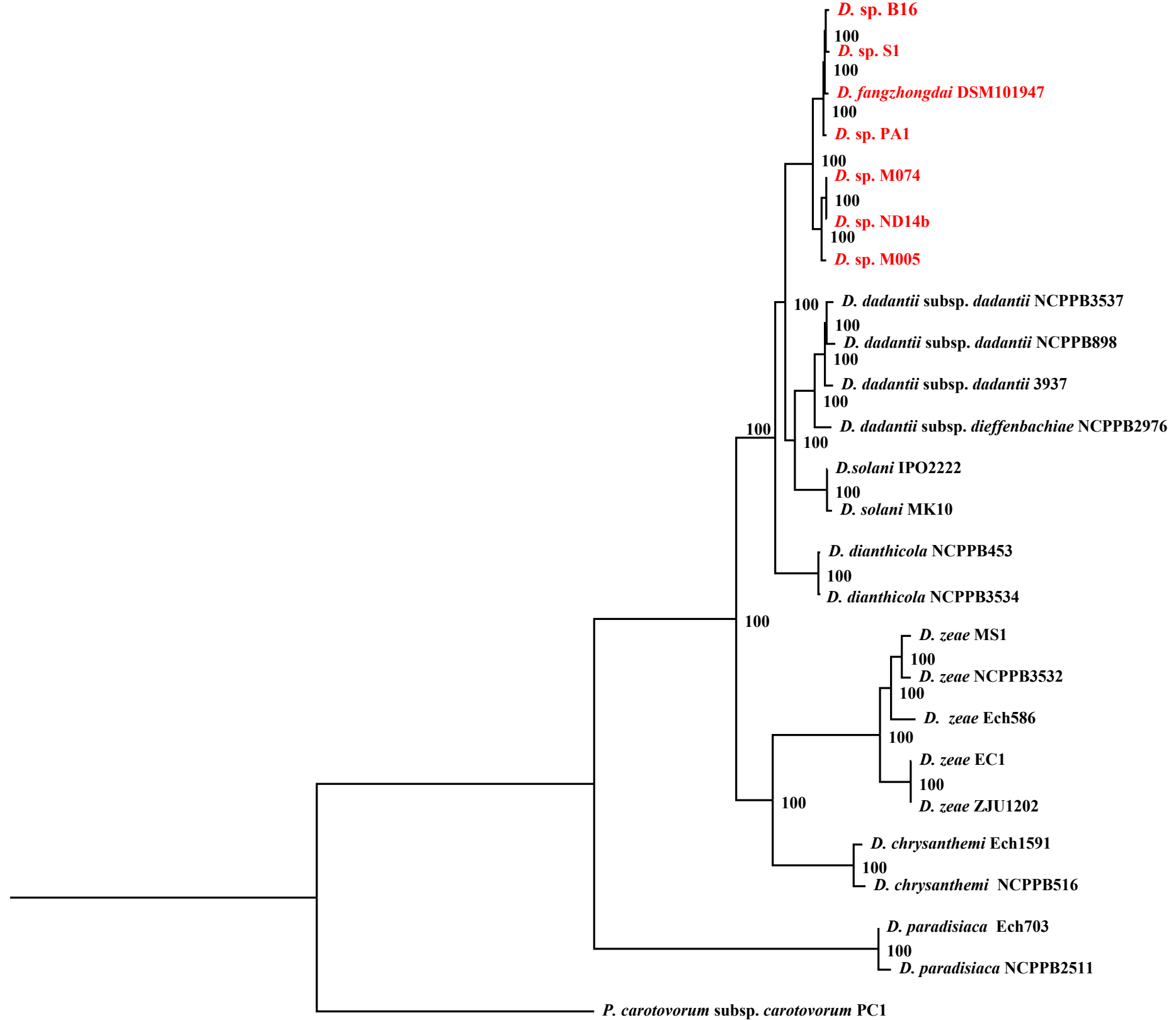

0.06

Supplement: Supplementary file 1 — Phylogenetic analysis of Dickeya strains based on complete or draft genome sequences. Orthologous groups present as a single copy in all of the analyzed Dickeya species were retrieved and their concatenated nucleic acid sequences used for phylogenetic analysis. (PDF 222 kb) [file 12864_2018_5154_MOESM1_ESM.pdf]

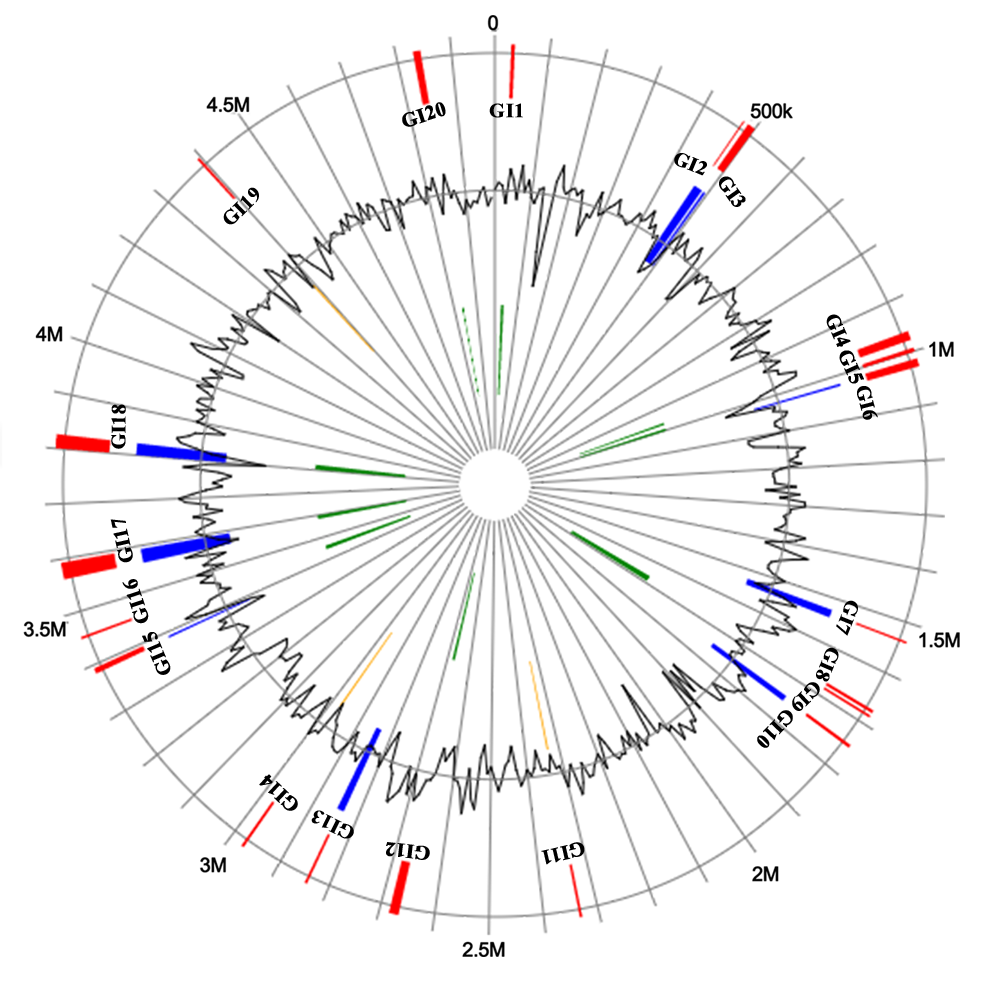

Supplement: Supplementary file 3 — GIs of D. fangzhongdai PA1 according to IslandViewer. This program integrates two sequence composition GI prediction methods, SIGI-HMM and IslandPath-DIMOB, and a single comparative GI prediction method, IslandPick. GIs predicted by one or more tools are highlighted in red on the outer circle and indicated by numbers. (TIF 4659 kb) [file 12864_2018_5154_MOESM3_ESM.tif]

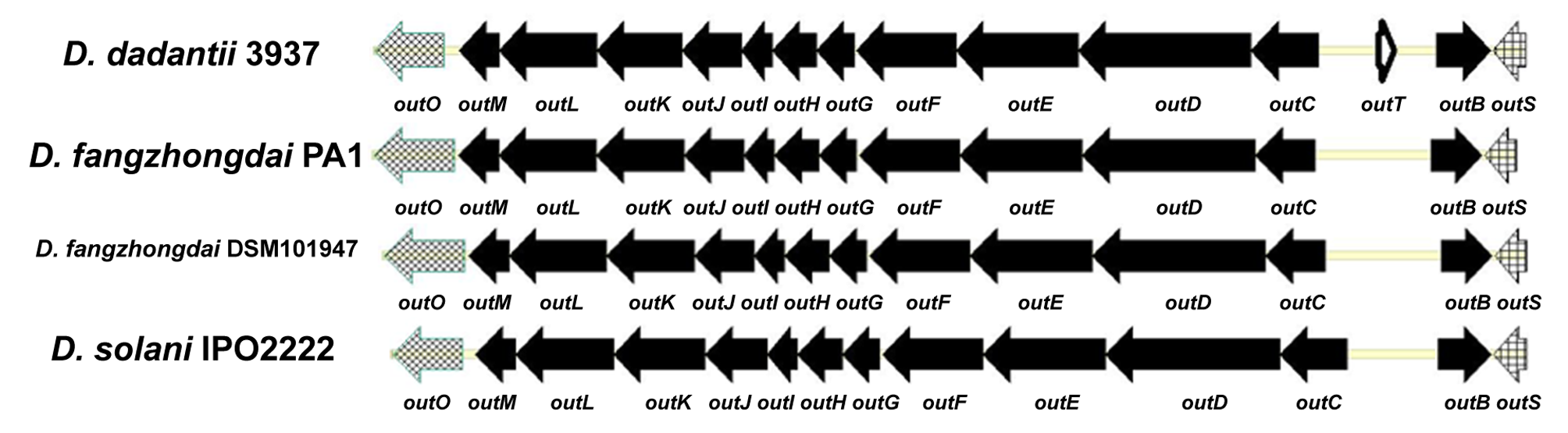

Supplement: Supplementary file 5 — Genomic organization of out-type T2SS in Dickeya strains. The out-type T2SS in D. fangzhongdai PA1 is at locus B6N31_15160–B6N31_15235. Blank boxes = T2SS component proteins; outS = gene encoding lipoprotein; outO = gene encoding prepilin peptidase. (TIF 498 kb) [file 12864_2018_5154_MOESM5_ESM.tif]

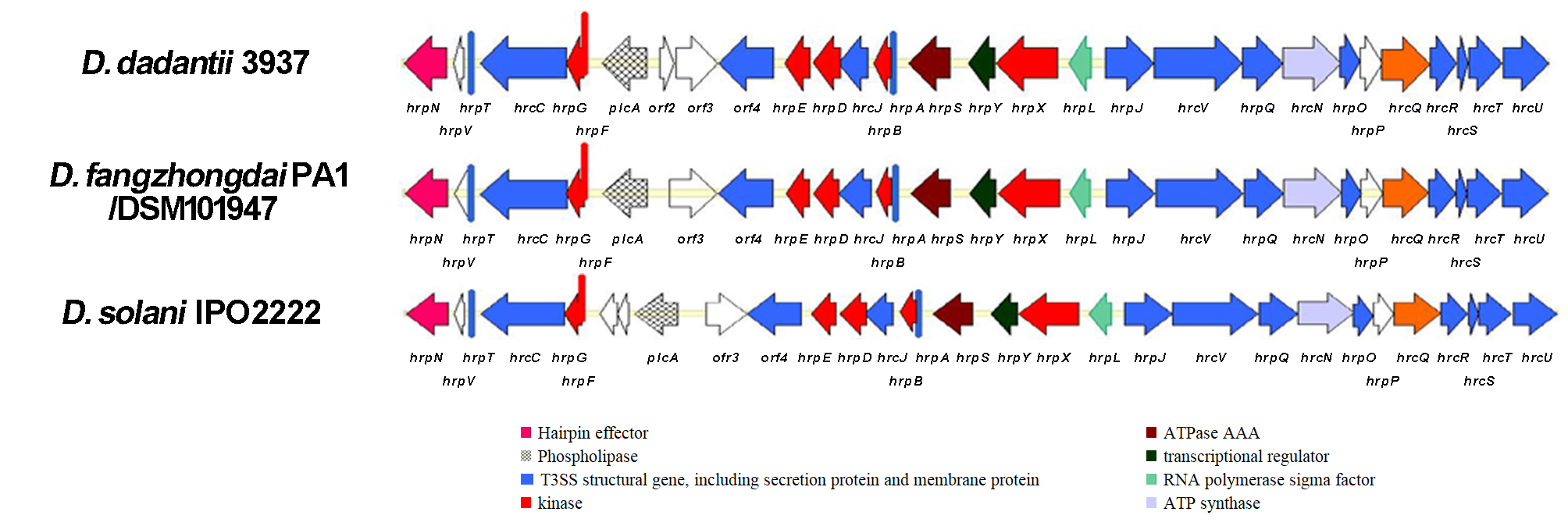

Supplement: Supplementary file 6 — Genomic organization of hrp-type T3SS in Dickeya strains. The hrp-type T3SS in D. fangzhongdai PA1 is at locus B6N31_12160–B6N31_12020. (TIF 2625 kb) [file 12864_2018_5154_MOESM6_ESM.tif]

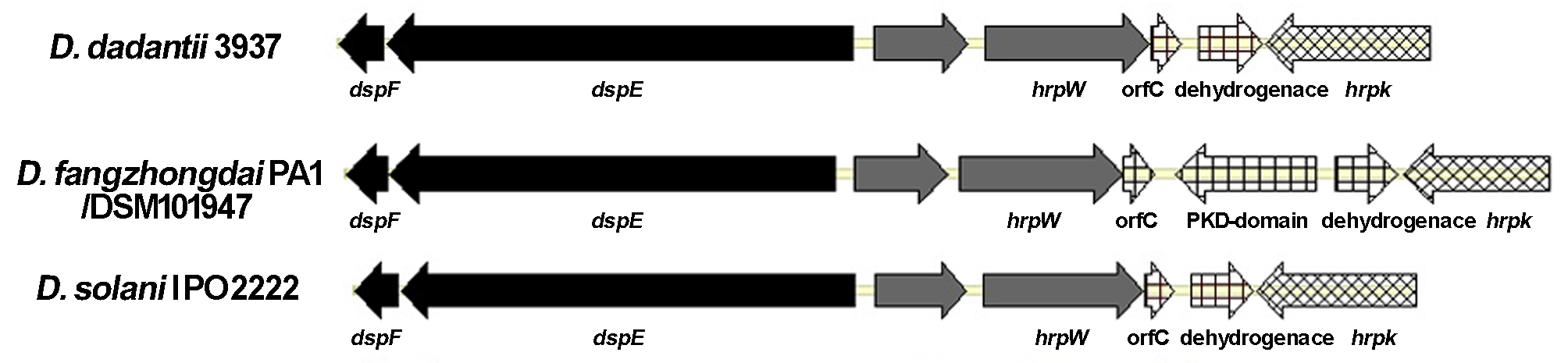

Supplement: Supplementary file 7 — Genomic organization of effector gene clusters of the T3SS in Dickeya strains. The effector gene cluster of the T3SS in D. fangzhongdai PA1 is at locus B6N31_11415–B6N31_11450. DspE/F = Avr family protein; HrpW = type III secreted protein; OrfC = DNA-binding protein; HrpK = pathogenicity locus protein. (TIF 1739 kb) [file 12864_2018_5154_MOESM7_ESM.tif]

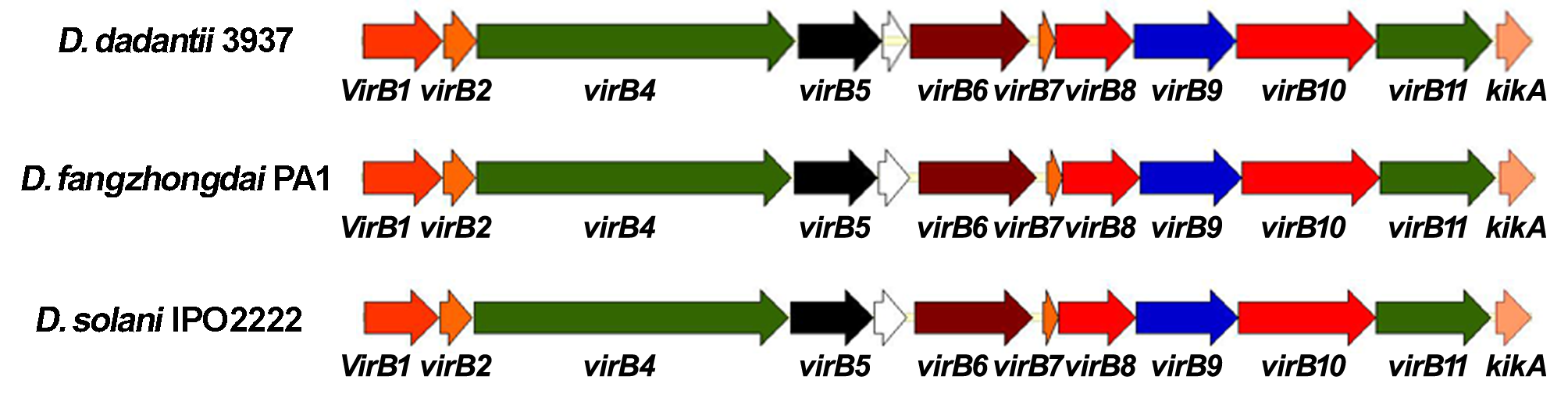

Supplement: Supplementary file 8 — Genomic organization of vir clusters of the T4SS in Dickeya strains. The vir cluster of the T4SS in D. fangzhongdai PA1 is at locus B6N31_08470–B6N31_08520. (TIF 1366 kb) [file 12864_2018_5154_MOESM8_ESM.tif]

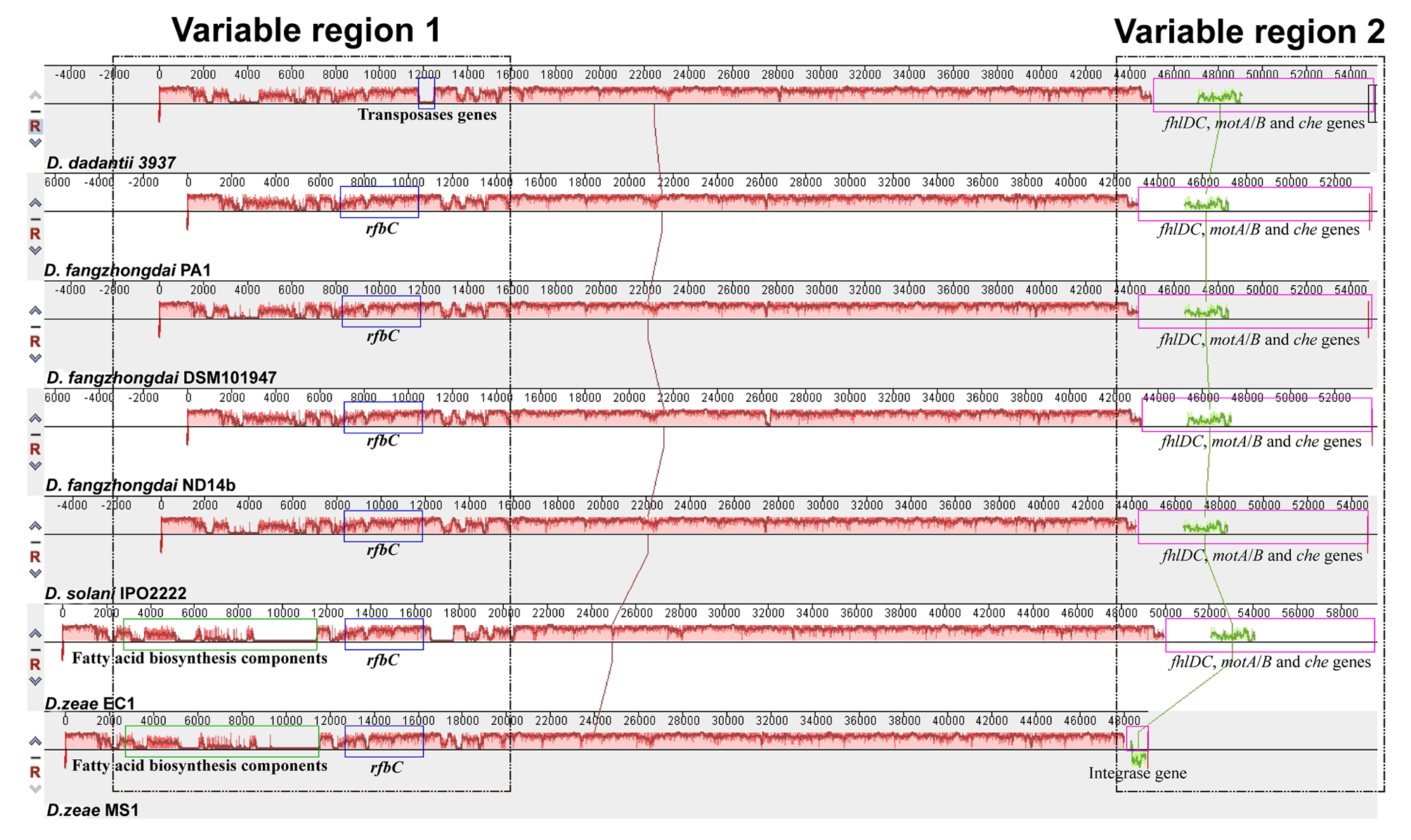

Supplement: Supplementary file 9 — Synteny analysis of Dickeya strains based on sequences of the flagellar-type T3SS. Strains analyzed included D. fangzhongdai PA1, DSM101947 and ND14b; D. dadantii 3937; D. solani IPO2222; and D. zeae EC1 and MS1. Nonconserved regions are indicated by different-colored frames. (TIF 7041 kb) [file 12864_2018_5154_MOESM9_ESM.tif]

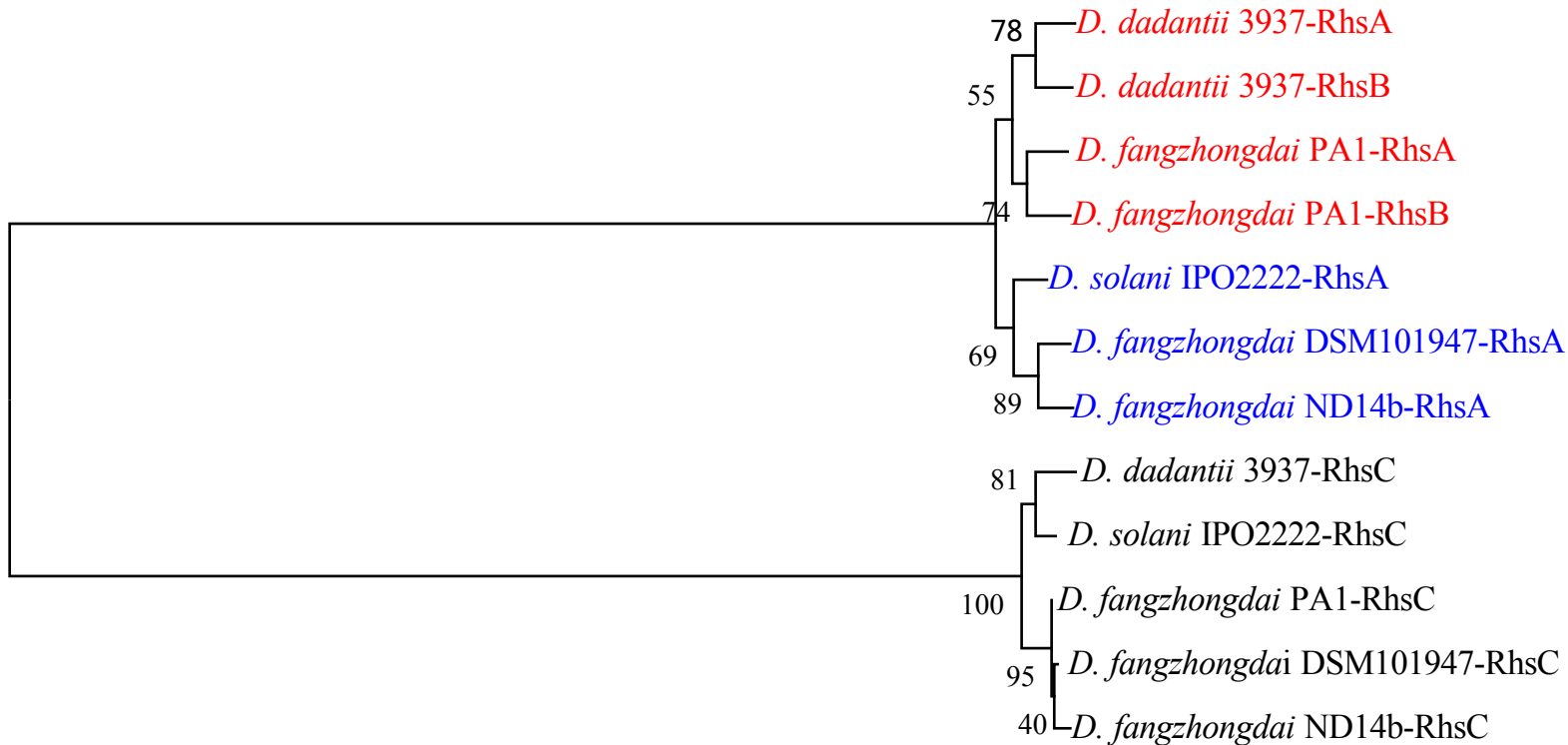

0.2

Supplement: Supplementary file 10 — Phylogenetic analysis of D. fangzhongdai, D. dadantii and D. solani strains based on the protein sequences of RhsA, RhsB and RhsC in T6SS. (PDF 156 kb) [file 12864_2018_5154_MOESM10_ESM.pdf]
